# Population Diversification in a Yeast Metabolic Program Promotes Anticipation of Environmental Shifts

**DOI:** 10.1371/journal.pbio.1002042

**Published:** 2015-01-27

**Authors:** Ophelia S. Venturelli, Ignacio Zuleta, Richard M. Murray, Hana El-Samad

**Affiliations:** 1 Division of Biology and Biological Engineering, California Institute of Technology, Pasadena, California, United States of America; 2 Department of Biochemistry and Biophysics, University of California San Francisco, San Francisco, California, United States of America; 3 The California Institute for Quantitative Biosciences, University of California San Francisco, San Francisco, California, United States of America; New York University, UNITED STATES

## Abstract

Delineating the strategies by which cells contend with combinatorial changing environments is crucial for understanding cellular regulatory organization. When presented with two carbon sources, microorganisms first consume the carbon substrate that supports the highest growth rate (e.g., glucose) and then switch to the secondary carbon source (e.g., galactose), a paradigm known as the Monod model. Sequential sugar utilization has been attributed to transcriptional repression of the secondary metabolic pathway, followed by activation of this pathway upon depletion of the preferred carbon source. In this work, we demonstrate that although *Saccharomyces cerevisiae* cells consume glucose before galactose, the galactose regulatory pathway is activated in a fraction of the cell population hours before glucose is fully consumed. This early activation reduces the time required for the population to transition between the two metabolic programs and provides a fitness advantage that might be crucial in competitive environments.

## Abstract 2

Detailed study of the dynamic response of yeast to combinations of sugars reveals an anticipatory population diversification strategy that allows rapid adaptation to shifts in environmental carbon source availability.

## Author Summary

To survive in resource-limited and dynamic environments, microbial populations implement a diverse repertoire of regulatory strategies. These strategies often rely on anticipating impending environmental shifts, enabling the population to be prepared for a future change in conditions. It has long been known that cells optimize nutritional value from mixtures of carbon sources, for example glucose and galactose, by sequential activation of regulatory programs that allow for metabolizing the preferred carbon source first before metabolizing the secondary carbon source. Using automated flow-cytometry, we mapped the dynamical behavior of populations simultaneously presented with a large panel of different glucose and galactose concentrations. We show that, counter to expectations, in populations presented with glucose and galactose simultaneously, the galactose regulatory pathway is activated in a fraction of the cell population hours before glucose is fully consumed. We demonstrate that the size of this fraction of cells is tuned by the concentration of the two sugars. This population diversification may constitute a tradeoff between the benefit of rapid galactose consumption once glucose is depleted and the cost of expressing the galactose pathway.

## Introduction

Microbial cells are continuously bombarded by diverse and changing combinatorial environmental stimuli. To survive and reproduce, a cell must accurately detect, assess, and selectively respond to these signals. Specifically, in competitive and unpredictable environments, cells need to constantly integrate information about the nature and quantities of nutritional substrates to scavenge maximum nutritional value [1]. Organisms that can balance the anticipation of future environmental shifts without sacrificing the rate of reproduction by excess metabolic burden exhibit a fitness advantage. However, metabolic strategies for achieving this balance have not been thoroughly explored.

Studies of the response of microbial cells to the availability of multiple sugars has a long history, starting with the seminal work of Dienert in yeast [2, 3] and Monod in bacteria [4, 5]. When presented with both glucose and galactose, microbial cells consume these carbon substrates in a sequential manner rather than simultaneously metabolizing both, resulting in two separate growth phases [5]. In the first phase, cells preferentially metabolize the sugar on which they can grow the fastest (glucose, in this case). Upon glucose depletion, cells transition to metabolizing the less preferred sugar after a “lag phase” in growth corresponding to the time needed to produce the necessary enzymes. Therefore, this response, classically known as “catabolite repression”, posits that the synthesis of the enzymes needed to metabolize the less preferred sugar is inhibited across the whole population. This inhibition is relieved by depletion of the preferred sugar, which triggers the diauxic shift. Crucially, in this model, the sequential consumption of the two sugars is generally attributed to the sequential expression of the enzymes needed for their metabolism [6].

Previous work has discussed regulatory strategies of microbial populations that constitute variations of the classical Monod model of uniform catabolite repression, and demonstrated that these strategies can facilitate adaptation to environmental change. Specifically, evolutionary tuning of the duration of the lag phase has been shown to be a crucial variable for fitness of microbial populations in fluctuating environments [7, 8]. For example, heterogeneity in the expression of the Lac operon in *Escherichia coli* has recently been shown to modify the growth rates of single cells during the transition from glucose to lactose metabolism [9]. Furthermore, evolution of *E. coli* in environments with mixtures of carbon sources has been shown to trigger genetic mutations that produce phenotypic population diversification as a consequence of key trade-offs in carbohydrate metabolism [10]. Similarly, yeast cells that were evolved in cycling maltose and glucose environments displayed reduced catabolite repression and enhanced cell-to-cell variability in the gene expression of the maltose pathway (MAL) in mixed environments with maltose and glucose [8]. Interestingly, the evolved strains that displayed salient heterogeneity in MAL gene expression across the population also had a lower growth rate on glucose and a reduced lag phase upon shift to maltose media.

Galactose is a less preferred sugar than glucose for the yeast *Saccharomyces cerevisiae*, and its metabolism requires activation of the galactose gene regulatory network (GAL). The GAL pathway is a well-studied circuit that includes a set of regulatory genes (Gal1p, Gal2p, Gal3p, Gal80p, and Gal4p) for sensing and signaling and enzymatic genes (Gal1p, Gal7p, and Gal10p) that transform galactose into glucose-6-phosphate as an entry point for glycolysis (S1 Fig.). Previous studies have shown that the GAL pathway can exhibit two stable gene expression states (ON and OFF) in a uniform environment in response to intermediate concentrations of galactose [11–14], and this bistability was demonstrated to be dependent on the Gal1p and Gal3p positive feedback loops [12, 14]. However, the gene expression dynamics of the GAL pathway in response to combinatorial glucose and galactose inputs and the relationship between the gene expression response and the diauxic shift have not yet been carefully characterized.

In this work, we demonstrate that while *S. cerevisiae* cells indeed undergo sequential sugar consumption in the presence of combinations of glucose and galactose, the synthesis of the enzymes needed for the metabolism of galactose is not necessarily sequential. Specifically, we find that for a large combinatorial space of glucose-galactose inputs, a subpopulation of cells arises where the galactose transcriptional program is induced hours before the depletion of glucose. We demonstrate that this heterogeneous strategy is beneficial for rapid growth during the metabolic transition from glucose to galactose. These data suggest that the response of microorganisms to combinatorial environments may frequently involve diversification of phenotypes across a population. Furthermore, this strategy integrates direct environmental sensing with an anticipation of future environmental shifts. As such, it constitutes an elaboration on bet-hedging mechanisms, which often rely on stochastic fluctuations to produce subpopulations with different phenotypes without a dominant input from the environment [15].

## Results

**Figure 1:**
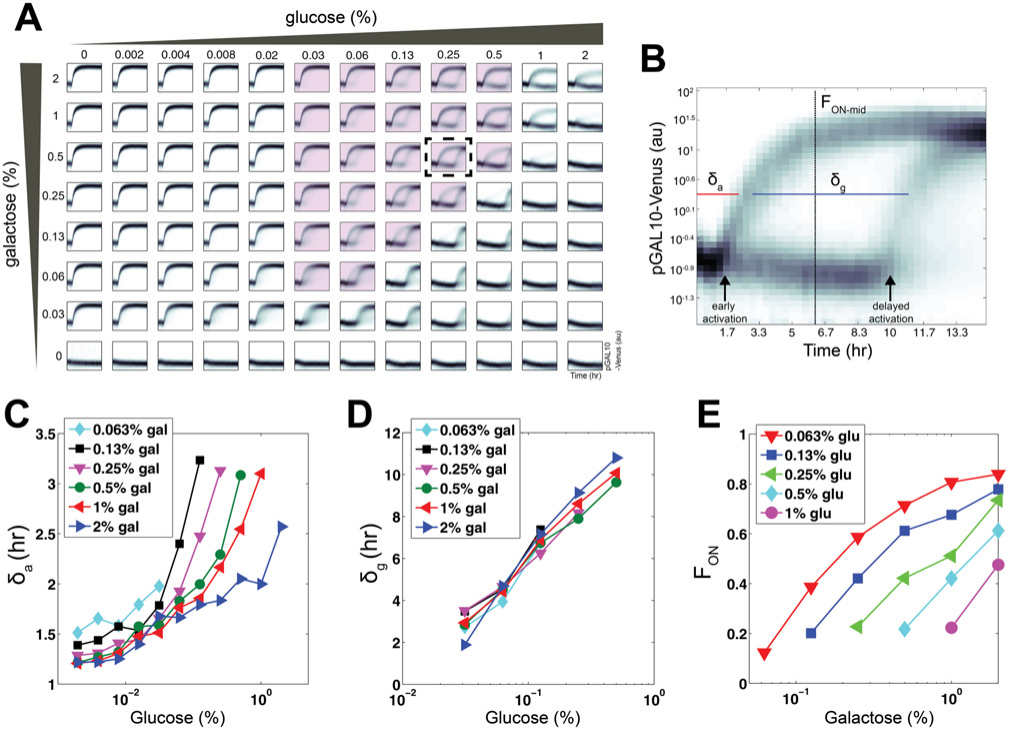
Dynamic responses of the GAL pathway to combinatorial inputs of glucose and galactose. **(A)** Single-cell fluorescence distributions of pGAL10-Venus as a function of time in wild-type haploid *S. cerevisiae* (W303) obtained using automated flow cytometry for a wide range of glucose and galactose concentrations. In each subplot, the *x*-axis is time and the *y*-axis is fluorescence, whose range is as displayed in panel **B**. Dashed box indicates the condition shown in panel **B**, and highlighted conditions were used to quantify the duration of bimodality (δ_g_) in panel **D. (B)** pGAL10-Venus flow cytometry distributions as a function of time highlighting transient bimodality (bottom) for cells induced with 0.25% glucose and 0.5% galactose. δ_a_ represents the response time of the early activated subpopulation, δ_g_ represents the duration of bimodality for highlighted conditions in panel **A** and F_ON-mid_ denotes the fraction of cells in the ON state at the midpoint of the transient bimodal region. **(C)** Relationship between initial glucose levels and δ_a_ for a range of initial galactose concentrations. **(D)** Relationship between initial glucose concentrations and δ_g_ for different initial galactose levels. **(E)** Relationship between initial galactose levels and F_ON-mid_ for different initial concentrations of glucose. Data for panel **A** can be found in S2 Data and **C**–**E** can be found in S1 Data.

We studied the time-resolved response of a population of yeast cells to combinatorial inputs of glucose and galactose using an automated flow cytometry setup that measures gene expression approximately every 20 min for 14 h (Fig. 1A) [16]. This technology enabled us to measure the galactose (GAL) pathway activity dynamics in single cells using the epimerase *GAL10* promoter (pGAL10) driving Venus (Yellow Fluorescent Protein, YFP) and to dissect the quantitative growth patterns of the microbial culture. The culture was maintained in exponential phase prior to transfer to a microtiter plate. The microtiter plate was inoculated at low cell density and diluted every 20 min for 3–6 h with fresh media while shaking at 30°C (see Materials and Methods). This period of growth and dilution before induction with the mixed sugar stimulus ensured that the growth history of the culture did not significantly influence the response of the cell population (S2 Fig.).

For sufficiently low glucose concentrations, pGAL10 induced as a single monomodal distribution. By contrast, pGAL10 did not activate over the course of the experiment for glucose concentrations significantly higher than those of galactose. These behaviors recapitulate previously observed phenotypes [17, 18]. However, for a large spectrum of combinatorial glucose-galactose inputs aggregating around the regime of equal concentration of these two sugars, we observed the emergence of a bimodal gene expression response in which only a fraction of the population induced pGAL10 (Fig. 1A, B). A single time point measurement has previously observed this bimodality in the GAL system for a mixture of glucose and galactose [11]. However, our dynamic measurements reveal that this bimodality is transient since the cohort of OFF cells uniformly switched ON following a delay. The promoters of the galactokinase *GAL1* (pGAL1), permease transporter *GAL2* (pGAL2) and transferase *GAL7* (pGAL7) exhibited similar gene expression patterns, indicating that transient bimodality is a general feature of the GAL pathway in response to a mixture of glucose and galactose (S3 Fig.).

Our flow cytometry data did not show a significant population of cells with intermediate fluorescence levels for bimodal populations and for a given dual-sugar input, the fraction of GAL ON cells did not change significantly over time in the bimodality region (highlighted box in S4A, B Fig.). Furthermore, previous studies have demonstrated that the GAL system can only exhibit stochastic transitions between states in the absence of the *GAL80* negative feedback loop but not in wild type [19]. To test for stochastic switching during the bimodal regime, we sorted cells induced with 0.25% glucose and 1% galactose into GAL ON and GAL OFF populations using pGAL10 expression following 4 h of induction (S5A Fig.). We then transferred the sorted subpopulations into one of three environments: (1) 0.25% glucose, (2) 1% galactose, or (3) a mixture of 0.25% glucose and 1% galactose (S5B, C Fig.). The ON cells gradually turned off over a period of 7.3 h in 0.25% glucose, and no detectable population of cells switched to the OFF state in either the galactose or the glucose and galactose mixture environment (S5C Fig.). These data strongly suggest that there is no stochastic switching from the GAL ON to the GAL OFF states. The GAL OFF cells remained in an OFF state in the environment with 0.25% galactose and activated the GAL pathway over a period of approximately 3.5 h in 1% galactose. However, we observed that a fraction of OFF cells induced pGAL10 when transferred into the mixed sugar condition. These cells are likely deriving from an intermediate population prior to the sorting process, since time-lapse microscopy measurements of the gene expression of single cells grown in microfluidic devices (CellASIC, see Materials and Methods) did not display stochastic switching between the ON and OFF states for a period of 6 h when induced with 0.25% glucose and 1% galactose and then switched to an environment containing 0.1% glucose and 1% galactose (*n* = 8 OFF or ON colonies shown in S6A, B Fig.). However, further experiments are needed to categorically rule out the possibility of stochastic switching from the GAL OFF to the GAL ON state before glucose depletion.

Using a Gaussian mixture model (GMM) to deconvolve the two populations (see Materials and Methods), we quantified three measures of the response: the time to early activation for conditions with a detectable early activated population (δ_a_), the delay between early and late activation for conditions with transient bimodality (δ_g_, highlighted panels), and the fraction of ON cells quantified at the midpoint between the half-max of the early and delayed activation responses (F_ON-mid_) (Fig. 1B, see Methods). δ_a_ was modestly increased by glucose and reduced by galactose (Fig. 1C). By contrast, δ_g_, showed a substantial linear increase as a function of initial glucose (highlighted panels in Fig. 1A and Fig. 1D). However, δ_g_ was not significantly modified by the initial galactose concentration (S7A Fig.). F_ON-mid_ significantly increased with the initial galactose level and was reduced by the initial glucose concentration for any given concentration of galactose (Fig. 1E). δ_g_ and F_ON-mid_ were modified in a set of mutants including regulators of the GAL pathway and glucose repression, suggesting that these phenotypes are modulated by a complex molecular program involving many factors (S1 Text and S8A–D Fig.).

To probe the dependence of this phenomenon on growth history, we compared the response of populations from stationary and exponential phase to a range of glucose and galactose inputs. Both conditions displayed a transient bimodal response, but stationary phase cells displayed a higher fraction of ON cells than cells deriving from exponential growth phase for any given sugar combination (S9 Fig.). Bimodality was also present in populations with different carbon source histories including YP alone, YP + 3% glycerol and YP + 3% ethanol. However, F_ON_ was larger for cells grown in glycerol and ethanol than cells grown in YP alone (S10 Fig.). These data indicate that transient diversification of the population into two expression states in response to dual-sugar inputs is a robust phenotype in our strain that persists for a range of growth histories. The quantitative properties of the transient bimodal response are, however, fine-tuned by population history.

**Figure 2:**
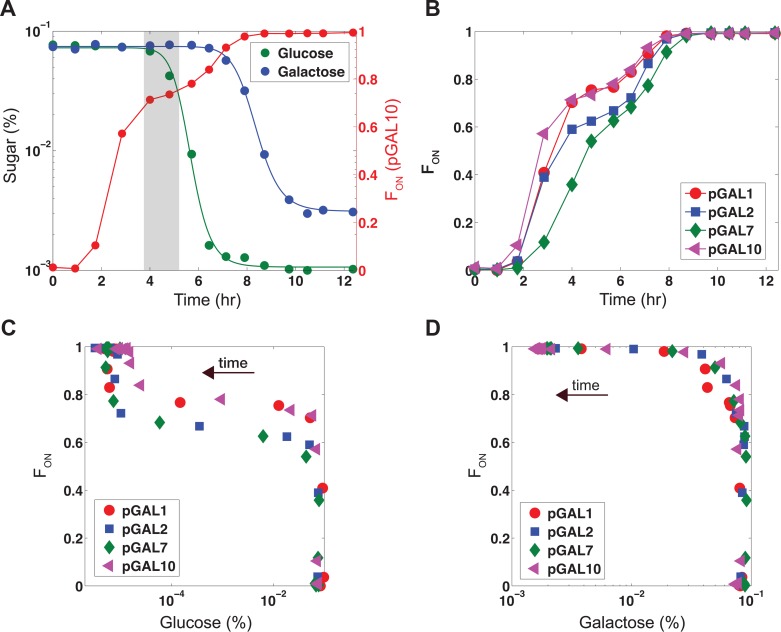
Glucose is consumed before galactose despite the presence of a subpopulation of cells with an active GAL pathway. Wild-type cells were exposed to 0.1% glucose and 0.1% galactose simultaneously at time zero. **(A)** Representative dynamic measurements of glucose, galactose, and the fraction of ON cells (F_ON_) for wild-type expressing p*GAL10-*Venus. Highlighted box indicates plateaued region. The lines through the glucose and galactose data represent fitted Hill functions. **(B)** F_ON_ as a function of time as scored by p*GAL1*, p*GAL2*, p*GAL7*, and p*GAL10* promoter fusions to Venus. **(C)** Plot of F_ON_ scored by either p*GAL1*, p*GAL2*, p*GAL7*, or p*GAL10* versus glucose concentration. **(D)** Plot of F_ON_ scored by either p*GAL1*, p*GAL2*, p*GAL7*, or p*GAL10* versus galactose concentration. Data can found in S1 Data.

The existence of a subpopulation of cells in which the galactose transcriptional pathway was active in the transient bimodality regime suggested that the population might be consuming galactose concurrently with glucose. To test this hypothesis, we measured glucose, galactose, and the fraction of ON cells (F_ON_) as a function of time in response to 0.1% glucose and 0.1% galactose, a condition in which the population exhibits a bimodal response (see Materials and Methods). Our data recapitulated the known sequential order of sugar utilization, with glucose being depleted before appreciable galactose consumption (Fig. 2A). Interestingly, F_ON_ scored by pGAL10, pGAL1, pGAL2 or pGAL7 increased immediately following the dual-sugar stimulus and transiently plateaued before the cells consumed the available glucose (Figs. 2A, B and S3 and S4). The initial concentration of glucose determined the duration of this plateau (S4C Fig.). F_ON_ underwent a second increase to approximately 100% precisely at the time of total glucose depletion. Therefore, the timing of the delayed activation of the repressed subpopulation, and consequently the magnitude of δ_g_, were determined by the time of glucose depletion. In agreement with this hypothesis, δ_g_ was inversely related to the initial population size N_0_, which modifies the rate of sugar consumption (S7B, C Fig.). In addition, a population that received a first step of glucose and galactose, followed by an additional step input of glucose after 5 h, had a significantly larger δ_g_ than a population that received only the initial dual-sugar input, further corroborating the fact that δ_g_ is tuned by the concentration of glucose (S7D, E Fig.). In contrast to δ_g_, F_ON-mid_ was approximately equal in conditions that received one or two steps of glucose, suggesting that the second glucose input did not induce substantial switching between OFF and ON states (S7F Fig.).

Our results indicate that galactose was not significantly consumed for hours despite the presence of a substantial subpopulation of GAL ON cells, making glucose the major contributor to biomass production up to the point of glucose depletion. Furthermore, during the bimodal epoch following an input of 0.1% glucose and 0.1% galactose, only approximately 2.7% of galactose was consumed when 78% of glucose was depleted (Fig. 2D). The expected amount of galactose consumption by the population of GAL ON cells is significantly higher if these cells were fully consuming galactose based on a culture that received only 0.1% galactose (S11A–C Fig.). This lack of significant galactose consumption could be due to failure of galactose to permeate into the cell or a different mechanism by which inhibition of galactose metabolism does not require transcriptional repression of the GAL genes. Gal1p and Gal3p are the only known sensors of galactose and these proteins function intracellularly, suggesting that a sufficient amount of galactose was entering the cells to induce pathway activation [20]. Furthermore, we observed a significant induction of pGAL2, accumulation of the fluorescently tagged Gal2 permease in the activated subpopulation, localization of Gal2p-Venus to the membrane, and strong correlation between a Gal2 fluorescent protein fusion and pGAL10 in the presence of mixtures of glucose and galactose (S3 and S12 Figs.). We also found that level of Gal2p was not limiting for the activation of the GAL pathway using a TET inducible promoter to vary the concentration of Gal2p in a strain deleted for the endogenous GAL2 gene. In this strain, we assessed the fraction of GAL ON cells (as quantified using a pGAL10-Venus reporter) in response to simultaneous addition of 0.5% galactose and a range of glucose levels (S12D Fig.). We did not observe a significant dependence of the fraction of ON cells on aTc concentration, and hence on Gal2p levels. These data are consistent with the previous observation that the inhibition of galactose consumption in response to a glucose pulse occurs on a timescale faster than can be explained by changes in transcriptional regulation or protein degradation [21].

**Figure 3:**
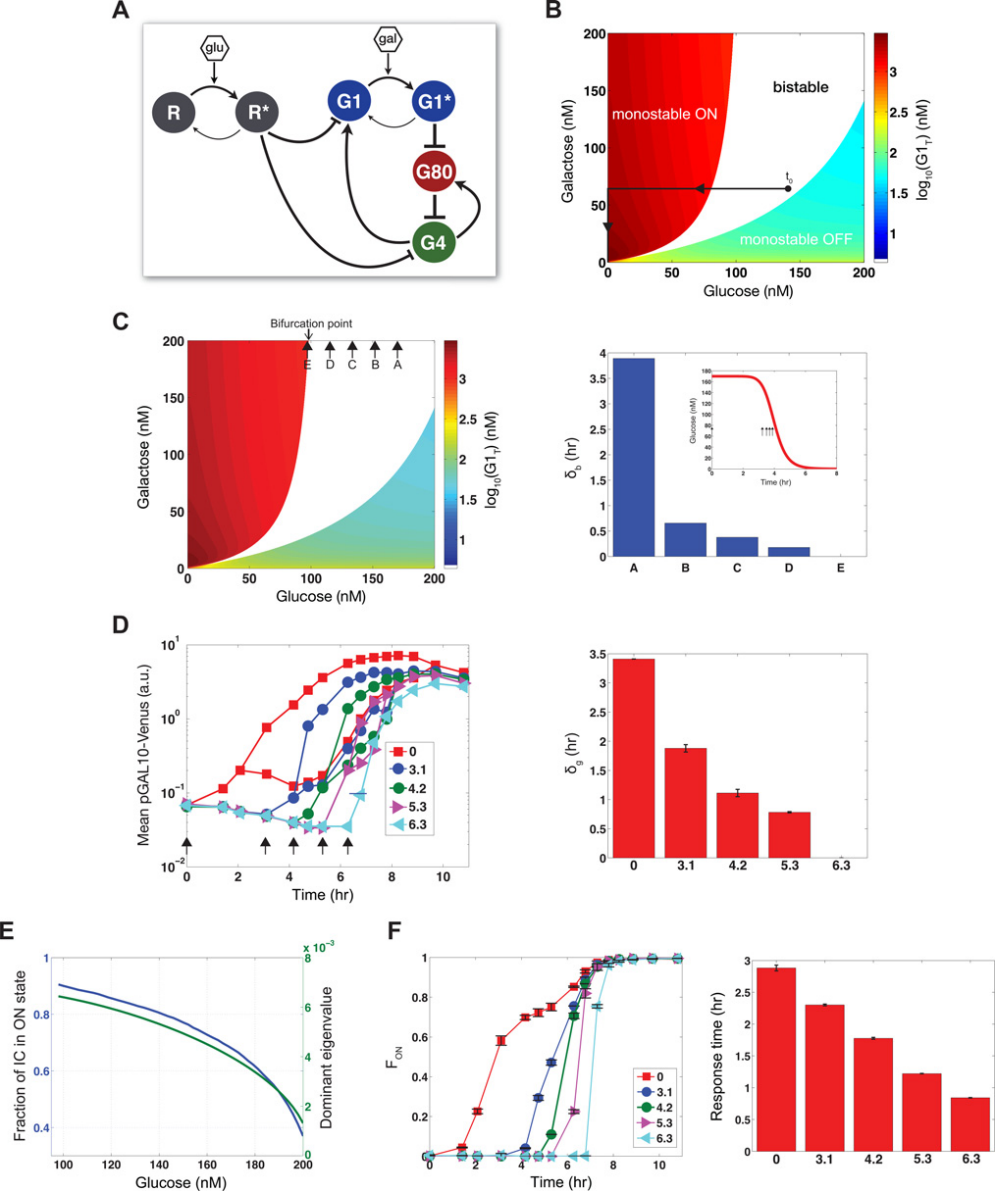
Computational model of GAL system with glucose and galactose inputs explains the origin of transient bimodality and predicts the dependence of the system on its inputs. **(A)** Schematic diagram of GAL circuit. Glu represents glucose and gal represents galactose. Pointed and blunted arrows indicate activation and repression, respectively. **(B)** Bifurcation diagram at steady-state. Bistability is represented by white and colored regions denote monostability. Total concentration of G1 is denoted by G1_T_. Time zero is indicated by t_0_ and solid lines highlight a model trajectory. **(C)** Bifurcation diagram at steady-state (left). Arrows denote the addition of galactose at different times to a system that received glucose at t_0_. δ_b_ is computed as the time required for glucose to decay to the bifurcation point threshold (right). **(D)** Mean expression levels of ON and OFF subpopulations for the delayed galactose experiment (left) and duration of bimodality (δ_g_) (right). Arrows indicate the time of the galactose stimulus. **(E)** The fraction of initial conditions (IC) and the dominant eigenvalue for the ON equilibrium state as a function of glucose for 150 nM galactose. **(F)** Experimental measurements of F_ON_ as a function of time in the galactose step experiment. Response time of F_ON_ for each condition (right). Error bars indicate one standard deviation (s.d.) from the mean of two replicates. MATLAB code for panel **B** can be found in S2 Data and data for **B**, **D**, and **F** can be found in S1 Data.

To understand how the structure of the GAL regulatory network could generate the observed transient bimodality in response to dual-sugar inputs, we constructed a simplified mathematical model of this circuit based on canonical knowledge about the galactose system (equations are described in the S1 Text). In our model, the galactose input activates the signal transducer Gal1p (G1) forming G1*, which inhibits the repressor Gal80p (G80) from sequestering the transcriptional activator Gal4p (G4), thus leading to GAL gene activation (Fig. 3A). The inhibition of G80 liberates G4 to induce expression of G1 and G80, establishing a positive and negative feedback loop. Since glucose has been shown to reduce the activity of the GAL system, we coupled this model to an input of glucose [22]. We modeled GAL repression by glucose assuming that a repressor R (such as Mig1), can be activated by the glucose signal forming R*, which can then repress the promoters of *GAL1* and *GAL4*. R links the glucose input to GAL repression, an additional feature to several previous models of the GAL system [12–14] that is well supported by literature [22–25] and follows a previous study that similarly simplified the complexity of the glucose repression network into a single module [26].

The GAL network has been shown to exhibit memory of galactose and glucose exposure, suggesting bistability as the source of bimodality in this system [14, 27]. A salient qualitative feature of our mathematical model is that it can undergo a bifurcation from monostability to bistability as a function of its two inputs: glucose and galactose (Fig. 3B). In response to low-glucose and high-galactose inputs, the model exhibits one steady-state, corresponding to the experimental ON state (high total G1 levels). For high glucose and low galactose inputs, the only steady-state corresponds to the OFF state (low total G1 levels). Similar concentrations of the two inputs produce two stable steady-states that correspond to the bimodality observed in the experiments. We also considered the possibility that glucose influences the GAL pathway through modulation of the growth rate, leading to different dilution rates of the proteins in the GAL network. However, such a model lacking an explicit repressor could not robustly recapitulate our data (S13 Fig., and equations are described in the S1 Text).

The glucose repressor model predicts the emergence and disappearance of bistability in the GAL system as a function of time for a given dual sugar input. By assuming that the system traverses a series of quasi–steady-states as a function of decaying sugar concentration (highlighted panels in Fig. 1), a given model trajectory crosses through a region of bistability, which is then transformed to monostability as glucose drops below a critical threshold (bifurcation point) due to cellular consumption (representative trajectory in Fig. 3B). This transition from bistable to monostable behavior at the glucose bifurcation point corresponds to the synchronized delayed activation of the repressed cohort of cells. The observed monomodal activation for sufficiently low glucose concentrations (left of highlighted panels in Fig. 1B) and significantly delayed activation (right of highlighted panels in Fig. 1B) are also explained by the model (S14A, B Fig.). Furthermore, the model indicates that if the glucose concentration were maintained above its value at the bifurcation point, for example by replenishment of glucose (Fig. 3C), then the window of time where bistability exists in the system would be extended. This is precisely the case since cultures that received an initial pulse of glucose and galactose followed by a second pulse of glucose exhibited bimodality for a longer period of time compared to a culture that received only the initial sugar mixture (S7D, E Fig.).

In addition to explaining a potential origin of transient bimodality, the model made predictions about different features of the system. First, the model had predictions about the role of feedback loops. Removing the *GAL80* feedback loop in the model augmented the range of glucose and galactose concentrations that produced bistability. This prediction was qualitatively consistent with our data showing that the range of glucose and galactose inputs that produced experimental bimodality was expanded in a strain lacking the Gal80p feedback loop (S15 Fig.). We also predicted using the model that irrespective of the identity of the negative regulator R, a reduction in repression strength by decreasing the binding affinity of R to the promoters of G1 and G4 would shift and contract the window of bistability (S14C, D Fig.). Consistent with this expectation, a mutant of the glucose-dependent negative regulator Mig1 (representing a component of R in the model) which has a reduced affinity for the Cyc8-Tup1 complex and therefore a diminished capability for glucose repression exhibited a smaller δ_g_ than wild type (S8B-1 Fig.). Halving the dosage of the Cyc8-Tup1 repression complex similarly exhibited a smaller δ_g_ than wild type (S8B-2 Fig.) [28].

The bifurcation hypothesis provided by the model also implied that the amount of time required for glucose to decrease to a threshold concentration corresponding to the bifurcation point in the system (δ_b_) should decrease if galactose is added at different times following the glucose input (rather than concomitantly with glucose) (S3C Fig.). This increasing delay in the galactose input signifies a decreasing glucose concentration in the culture due to cellular consumption at the time of galactose addition, and hence a reduced window of time for bistability. The delayed activation response corresponds to the loss of bistability as glucose crosses a threshold bifurcation point. Hence δ_g_ and δ_b_ reflect similar properties of the system.

To experimentally test this prediction, we applied a step input of 0.1% galactose at different times to a set of cultures that had all received 0.1% glucose from time zero. Galactose was added at 0, 3.1, 4.2, 5.3, and 6.3 h following the glucose stimulus to different cultures (arrows in Fig. 3D). Matching the trend of decreasing δ_b_ in the model (Fig. 3C), bimodality emerged at the time of the galactose input and δ_g_ contracted and eventually disappeared with the increased delay in this input (right panel in Fig. 3D and S16 Fig.).

Finally, the model indicated that the response time of the system to transition from the OFF to the ON state decreases as glucose decays. The system’s response time is dictated by both the domain of attraction and the magnitude of the dominant eigenvalue of the ON steady-state (S1 Text), which both increase as glucose decreases (Fig. 3E). Therefore, the response time of the fraction of ON cells should decrease in the delayed galactose experiment. Corroborating this insight, our experimental data demonstrated a decrease in the response time of F_ON_ with the increase in the delay of the galactose input (Fig. 3F, G). Therefore, our model provides a framework that explains the transition of the GAL system between different phenotypic modes and demonstrates the modulation of the quantitative properties of this network by its environmental inputs.

**Figure 4:**
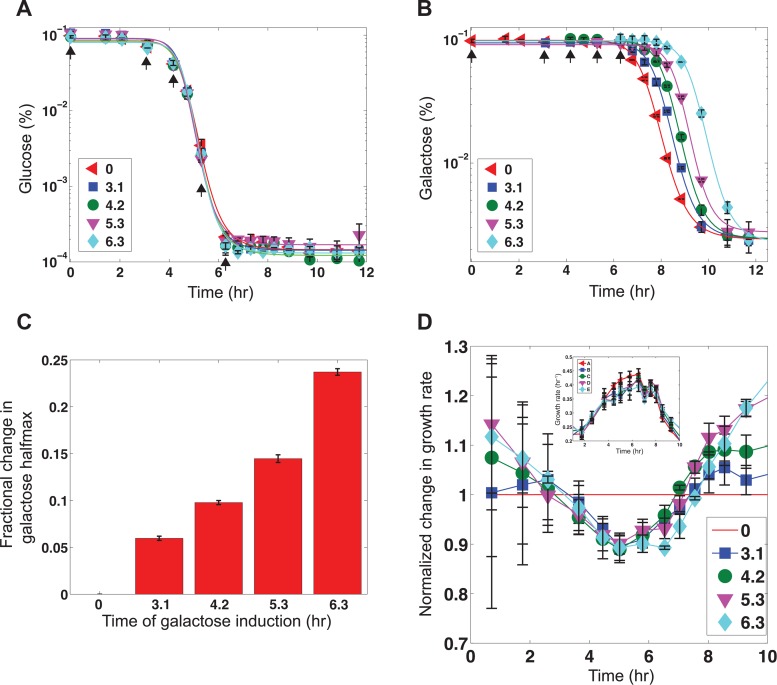
Early GAL pathway induction establishes a growth advantage during carbon source switch. Measurements of glucose, galactose, and growth rates for delayed galactose experiment in which 0.1% galactose was added to a set of cultures at different times that had all received 0.1% glucose from time zero. Arrows indicate the time of the galactose stimulus. **(A)** Glucose concentrations as a function of time for each condition. **(B)** Galactose concentrations as a function of time for each condition. Lines represent fitted Hill functions. **(C)** Fractional change in the half-max of the galactose decay curves for each condition relative to the culture that received glucose and galactose simultaneously at the beginning of the experiment (0). **(D)** Normalized growth rates of cultures that received galactose at 3.1, 4.2, 5.3, and 6.3 h after glucose to that of a culture that received galactose and glucose simultaneously (red line). Un-normalized growth rates for each condition (inset). Error bars represent one s.d. from the mean of two replicates. Data for this figure can be found in S1 Data.

We next probed the physiological impact of the observed anticipatory induction of the GAL regulatory program in advance of galactose consumption by analyzing the relationship between the timing of GAL pathway activation and the population’s growth rate and metabolism. To do so, we quantified the concentrations of glucose, galactose, and growth rates for the different cultures that were subjected to delayed galactose inputs in the experiment described above. Glucose decayed at a similar rate irrespective of when galactose was added (Fig. 4A). By contrast, galactose consumption was delayed for cultures that received galactose at 3.1, 4.2, and 5.3 h compared to the culture that received glucose and galactose simultaneously at time zero despite the fact that not all glucose was depleted at the time of galactose addition (Fig. 4B, C). Furthermore, the delay in galactose consumption was increased commensurately with the delay in galactose administration. Notably, during the metabolic shift between carbon sources, the population that received galactose simultaneously with glucose exhibited a transient growth rate advantage, reaching approximately 15% compared to the population that received this sugar after a 6.3 h delay (Fig. 4D and S17 Fig.). Since the growth rate is proportional to the current size of the population in exponential phase, the significance of this fitness difference increases with each cell generation. Overall, the delay in galactose input caused a monotonic increase in the transient growth defect, which was manifested as an increase in the “lag” time between the two phases of growth documented by Monod (S17A Fig.) [4]. Growth rate measurements indicate that the presence of galactose did not benefit the population of cells until total glucose depletion (S17 and S18 Figs.). Taken together, these data indicate that the induction of the GAL pathway many cell generations before these genes are required provides a transient fitness advantage during the shift between carbon sources.

This beneficial pre-emptive induction of the GAL pathway genes only occurs in a subset of the population. To investigate the tradeoffs that might motivate this bimodal induction, versus a uniform strategy in which all the cells in the population pre-emptively but coherently induce the GAL pathway, we sought to control GAL gene expression independently of galactose. To do so, we used an estradiol inducible Gal4 chimera in a strain lacking endogenous Gal4p [29, 30]. In this strain, we could activate GAL gene expression on demand at specific times before glucose depletion in cultures subjected to a mixture of 0.1% glucose and 0.1% galactose at time zero (S19A Fig.).

Since the synthetic inducible system is not connected to the feedback structure of the natural circuit, GAL gene expression was monomodal (graded) as opposed to bimodal. In this strain, early activation of the GAL pathway generated a lower consumption rate of glucose compared to late activation, therefore revealing that constitutive GAL gene expression can inhibit glucose consumption (S19B Fig.). The expression level of pGAL10 induced by this synthetic system was very similar to the expression level of pGAL10 in the wild type (S19A Fig.). Therefore, this effect is not likely to be a consequence of over- or under-expression of the GAL genes. Constitutive induction of the GAL pathway through over-expression of Gal3p also reduced the glucose consumption rate (S20 Fig. and S1 Text). Together, these data highlight an important tradeoff that the system has to balance: induction of the GAL genes before they are required results in faster galactose consumption upon glucose depletion, facilitating the transition between carbon substrates. At the same time, wholesale induction of these genes across the entire population comes at the cost of a reduced rate of glucose consumption. In agreement with this observation, the GAL repressed subpopulation in the wild type had approximately 15% faster growth rate on average than that of the activated subpopulation in the transient bimodal region (S21 Fig. and S1 Text). However, in this bimodal regime, the glucose consumption rate of the whole population was not saliently reduced by GAL gene expression in a subpopulation of cells (Fig. 4A).

To further highlight the tradeoffs of the bimodal strategy adopted by the population in combinatorial carbon environments, we tracked the growth and gene expression of 13 single ON or OFF colonies over time induced with 0.25% glucose and 1% galactose using time-lapse microscopy in microfluidic chambers (CellASIC, see Materials and Methods). In a first experiment, cells were grown for 3 h in 0.25% glucose and 1% galactose and then switched to 1% galactose (S22 Fig.). The GAL ON cells grew faster following the abrupt switch to galactose media than the OFF cells, corroborating our flow cytometry results that demonstrated that pre-induction of the GAL genes hours prior to the metabolic transition provides a fitness advantage. During the initial 3 h in the combinatorial environment prior to the switch to galactose media, we could not detect a significant difference in the growth rates of the OFF and ON cells, likely because this period of time, which spans approximately two cell doublings, cannot reveal such difference. Instead, we hypothesized that a longer experiment and graded reduction in the glucose concentration is necessary to detect a difference in the subpopulation growth rates. To test this notion, we exposed a culture to a mixture of 0.25% glucose and 1% galactose for 3 h followed by a mixture of 0.1% glucose and 1% galactose for an additional 3 h (S6 Fig.). In the lower glucose environment, the OFF cells grew significantly faster than the ON cells demonstrating the fitness cost imposed on cells by the induction of their GAL program in an environment where these genes are not required (S6C Fig.).

## Discussion

In this work, we demonstrate that a combinatorial input of glucose and galactose triggers diverse regulatory states across a population of cells. This transient bimodality establishes the co-existence of two subpopulations of cells—one that prepares hours in advance for a future shift in carbon metabolism and a second that defers pathway activation over many cell generations until these genes are required upon glucose depletion. The fraction of cells that occupies each state is tuned by the dual-sugar mixture, standing in contrast to canonical models in which the output of a pathway is proportionally matched to the level of its inputs in all cells of the population [31, 32].

Anticipatory responses to environmental change have been documented in a number of cellular systems [33, 34]. For example, correlations between heat shock and low oxygen in the human gut are thought to cause *E. coli* to trigger both responses in the presence of any single one of these environmental cues [33]. Here, we show anticipatory induction of galactose utilization genes many hours before glucose depletion. A new study appearing in the same issue documents that early induction of the GAL pathway in the presence of both glucose and galactose is a general response of many *S. cerevisiae* isolates [35]. Consistent with our data showing that early synthetic induction of the GAL genes can shorten the lag phase upon glucose depletion, the Wang et al. study further confirms that in these strains, the length of the diauxic lag is strongly determined by how early the galactose pathway is induced. Similar conclusions were reached in a study of the maltose pathway in yeast [8], indicating that preemptive induction of metabolic programs might be a general strategy that microorganisms use to enhance their preparedness for depletion of a preferred nutrient in mixed-nutrient environments.

A second feature in our data is that for many combinations of mixed glucose and galactose concentrations, only a fraction of the population undergoes early induction of the GAL program. This fraction is further dependent on the concentration of the sugars. The GAL ON subpopulation grows slower than the GAL OFF subpopulation in the presence of glucose, suggesting that while early induction of the GAL program offers a delayed benefit by shortening the lag phase upon glucose depletion, it also incurs an immediate cost when glucose is present. Our data indicate that this cost is partially the result of a slowdown in glucose consumption. Similarly, the natural isolate with the earliest GAL induction (and shorter lag phase) in the Wang et al. study was found to exhaust glucose slower than the strain with delayed GAL induction (and longer lag phase) [35]. These observations point to a tradeoff in which populations balance loss of immediate fitness with future benefit.

There are two broad strategies by which organisms can strike this balance to adapt to the environmental statistics of their particular niche. First, they can tune the parameters of the early activation of the GAL program in the whole population, delaying or accelerating its time and/or its induction kinetics. Alternatively, as we show in this paper, they can implement a stochastic diversification strategy, akin to bet-hedging [15, 36, 37], whereby only a fraction of the population undergoes early GAL gene activation, therefore bearing the immediate cost in the presence of glucose and reaping its benefits upon glucose depletion. However, this fraction (and hence the cost and benefit balance) is dependent on the state of the environment (the concentration of glucose and galactose). As a result, the strategy can be viewed as an elaboration of bet-hedging resembling the proposed mechanism of “stochastic sensing” [1, 38, 39].

Intriguingly, the different natural isolates probed in the Wang et al. study exhibit an array of population behaviors, ranging from strains exhibiting early unimodal induction of the GAL program to others showing transient bimodality in gene expression across the population [35]. Furthermore, evolved yeast cells after cycling between maltose and glucose inputs exhibited an array of behaviors including “specialist” mutants that have higher fitness under the glucose-only condition at the expense of a longer lag phase, and “generalist” mutants with a shorter lag phase and increased fitness during transition between carbon sources, but at the expense of decreased fitness during growth on glucose [8]. Interestingly, some of the evolved strains displayed a bimodal expression of the MalS gene across the population, although this phenotype was not dynamically characterized. Therefore, we postulate that while early GAL gene induction is likely to be a general anticipatory strategy of glucose depletion, the timing of induction and degree of heterogeneity in gene expression across the population varies among different strains, possibly as a result of distinct selective pressures and environmental statistics [40].

We observed that GAL ON cells did not appreciably metabolize galactose until glucose depletion. Although our data do not pinpoint the exact mechanism by which galactose metabolism might be inhibited in GAL ON cells, several possibilities exist. Previous studies examining this issue to produce biotechnologically efficient yeast strains documented that glucose and galactose are consumed simultaneously in cells lacking the glucose kinase Hxk2p [41]. Furthermore, in *S. cerevisiae*, glucose was shown to block the maltose pathway (MAL) by a novel mechanism at the signaling level, which is also linked to Hxk2p [42]. Finally, all but one of the evolved “generalist” strains with the shorter lag phases in the New et al. study harbored mutations in HXK2, which led to reduced catabolite repression of the MAL genes [8]. It is therefore possible that inhibition by glucose proceeding though HXK2 can have both transcriptional (catabolite repression) and post-translational inhibitory components, and is general to both the GAL and MAL pathways. If this were the mechanism at play, then GAL gene expression and galactose metabolism would be at least partially decoupled in the cell. However, other mechanisms might also explain our observations. For example, our data in Fig. 2 show that the bimodal regime following a dual-sugar input is delayed in pGAL1 and pGAL10 versus pGAL2 and pGAL7. Therefore, it is possible that temporal ordering in the expression of the GAL genes provides a timer-like mechanism for galactose consumption. An intriguing outcome of this model is that different yeast strains or species can modulate this timing based on their niche requirement, hence possibly consuming galactose while glucose is still available. However, further studies are needed to unravel the intricate molecular mechanisms that shape the coupling of gene expression and metabolic decisions of cells and the fitness trade-offs involved in specialization to a single environment versus rapid adaptation to a shift in environmental conditions. It will be fascinating to systematically dissect the combinatorial space of different regulatory and metabolic strategies in other *S. cerevisae* strains and different microorganisms and link their adoption to specific challenges for survival and reproduction in complex competitive environments.

## Materials and Methods

### Yeast Strains

All yeast strains were derived from W303. All plasmids used in this study were derived from a set of yeast single integration vectors containing selectable markers and targeting sequences for the LEU2, HIS3, TRP1, and URA3 loci. These vectors were linearized by digestion with PmeI and transformed using standard yeast transformation techniques. The sequences for the GAL1, GAL2, GAL7, GAL10, MIG1, and ADH1 promoters were 646, 743, 729, 657, and 658 bp upstream from the start codons for these genes, respectively. The genotypes for these strains are listed in S3 Table. Several strains used in this study have been described previously [14].

### Growth Conditions for Flow Cytometry

Cells were grown at 30**°**C for all experiments. For all experiments except microscopy, cells were grown overnight in yeast peptone media (YP) containing 10 g/L yeast extract and 20 g/L peptone for approximately 12 h. This culture was diluted to an optical density (OD_600_) of approximately 0.05–0.1 and grown for an additional 5–6 h in a 10 ml volume (final OD_600_ ranged between 0.3–0.7). This single well-mixed culture was then diluted appropriately with fresh YP media into a 96-well microtiter plate to a final OD_600_ of less than 0.02 (500 μL volume). Cells were grown in the 96-well plate and diluted every 20 min by a liquid-handling robot with YP media for 2–4 h prior to induction with glucose and galactose for all experiments except the direct comparison of stationary and exponential phase cultures in S9 Fig. [16]. This growth and dilution period further ensured that the cell population was in exponential phase at the time of the sugar stimulus. For the experiments shown in S9 Fig., stationary phase cultures were grown for 26 h without dilution until saturation due to nutrient depletion in the media. Exponential phase cells were grown and diluted as explained above. An outgrowth period was not performed for this experiment. Further details about the flow cytometry automation can be found in [16].

### Flow Cytometry

Single-cell fluorescence was measured on a LSRII analyzer (BD Biosciences). A blue (488 nm) laser was used to excite YFP and emission was detected using a 530/30 nm filter. For each measurement, 1,000–20,000 cells were collected.

As described previously [16], a 500 µl culture volume was used in 96-well plate format for the automated flow cytometry measurements. For step-response experiments, a 30 µl sample was removed from the culture for measurement on the cytometer at each time point and 30 µl of fresh YP media containing the appropriate 1X concentration of glucose and galactose was used to maintain a constant culture volume. The sugar pulse experiments shown in S2B, S9 and S10 Figs. were performed by the addition of a range of glucose and galactose concentrations at the beginning of the experiment, and 30 µl of fresh YP media lacking the two sugars was added at each time point.

### Flask Measurements

A 60 ml culture volume was used for the flask experiments in which the sugar concentrations were quantified. Less than 5% of the total volume was removed over the course of the experiment to quantify the single cell fluorescence, sugar concentrations, and absorbance at 600 nm (OD_600_). OD_600_ was measured on a Nanodrop 2000c spectrophotometer (Thermo Scientific).

### Quantitative Analysis of Gene Expression Dynamics

The ratio of YFP fluorescence to side scatter was used to quantify the total fluorescence per cell. Flow cytometry distributions were analyzed using a Gaussian mixture model algorithm (GMM, MATLAB) and each distribution was classified as either unimodal or bimodal, as described in [10]. The delay time δ_a_ was computed as the time required to reach the half-max of the mean of the activated subpopulation. δ_g_ was defined as the difference between the time required to reach the half-max of the mean of the activated and repressed subpopulations. The fraction of ON cells (F_ON_) was computed as the fraction of the cell population higher than a fluorescence threshold (10^−0.2^ a.u.) that corresponds to approximately the lowest density of single-cell fluorescence between the OFF and ON expression states. F_ON-mid_ was quantified at the midpoint between the half-max of the activated (δ_a_) and repressed subpopulations. The response time was defined as the time to reach the half-max of F_ON_ (F_ON_ = 0.5). At each time point, individual cells were assigned to the OFF and ON states using the F_ON_ threshold on gene expression described above. The subpopulation growth rates were computed as the slope of a line fit to the log_2_ of the number of cells that accumulated in the OFF and ON states over time.

### Sugar Measurements

Glucose and galactose were measured using the Amplex Red glucose oxidase and galactose oxidase kits (Molecular Probes, Life Technologies). A Safire II plate reader (Tecan) was used to quantify the fluorescence. A standard of known concentration for each sugar was used to determine the quantitative relationship between the fluorescence and sugar concentration. The maximum concentration of sugar was in the linear range as shown in S11 Fig.

### Microscopy Experiments

Time-lapse microscopy was performed using the Y04C plates from CellASIC ONIX microfluidic platform (EMD Millipore). For microscopy experiments, cells were grown overnight in minimal media containing all amino acids, yeast nitrogen base and 2% filter-sterilized raffinose. In the morning, cells were diluted to an OD_600_ of 0.1 in minimal media containing 1% galactose, 0.25% glucose and grown for 4 h prior to inoculation of the microfluidic chamber. For the diauxic shift experiment (S22 Fig.), the media also contained 2% raffinose. The mid-log phase cells (final OD_600_ was approximately 0.3–0.5) were sonicated briefly, centrifuged for 2 min at 2,000 rpm and then resuspended in a smaller volume of minimal media containing 1% galactose and 0.5% glucose such that the OD_600_ was equal to 0.8–1.5. 1µl of the concentrated cells was loaded into the Y04C microfluidic chamber and 200 µl of minimal media containing glucose and/or galactose was loaded into the flow wells. The chambers were loaded with 8 psi and media continuously flowed at 5 psi over the course of the experiment. Cells were imaged every 15 min at 30°C on a Nikon Ti-E equipped with a Perfect Focus System and a Coolsnap HQ2 CCD camera (Photometrics) located at the Nikon Imaging Center (UCSF). Imaging was performed using a Plan Apo 20x/0.25 and Plan Apo 40x/0.25 objectives.

### Cell Sorting

Wild-type cells were grown in YP media overnight. In the morning, the saturated culture was diluted to an OD_600_ of 0.1 and induced with 1% galactose and 0.25% glucose for 4 h at 30**°**C in YP media. After resuspending the cells in 1X PBS, they were sorted based on pGAL10 fluorescence using a FACS Aria II (BD Biosciences). 3e6 ON cells and 500,000 OFF cells were collected. Following the sort, the ON and OFF cells were centrifuged for 10 min at 2000 rpm. These cells were used to inoculate three 10 ml cultures containing 0.25% glucose, 1% galactose and 1% galactose and 0.25% glucose in YP media and were grown at 30**°**C. Samples were taken approximately every 40 min (S5B, C Fig.) for 7.3 h and the fluorescence was measured on an LSRII analyzer (BD Biosciences).

### Microscopy Data Analysis

For microscopy data, cell budding events over time were analyzed manually. Single-cell fluorescence was quantified in ImageJ using the ROI toolbox to track single cells over multiple frames. Single-cell fluorescence was defined as: integrated density − (area of selected cell × mean fluorescence of background). The population growth rates were defined as the finite difference of the natural logarithm of the OD_600_ measurement divided by the change in time in hours.

### Computational Modeling

We used custom code for mathematical modeling written in MATLAB (Mathworks) and Mathematica (Wolfram Research). Details about the model construction are provided in the S1 Text. The parameter values are listed in S1 Table and S2 Table. The domain of attraction of the ON steady state was defined as the fraction of initial conditions that were assimilated by the ON equilibrium point and was determined by randomly sampling 5,000 initial conditions using the Latin Hypercube Method [14]. A minimum and maximum bound on the concentration of each species was used based on the parameters of the model. The dominant eigenvalue was defined as the eigenvalue of smallest absolute value of the linearization at the ON equilibrium point.

## Supporting Information

S1 Data This file contains data for Figs. 1C–E, 2A–D, 3B, D, F, 4A–D and S2, S4, S6–S13, S15, and S17–S22 Figs. (XLSX)

S2 Data This file data for Figs. 1A, S2, and S3 and MATLAB code for generating Figs. 3B and S13B. (ZIP)

S1 Fig The galactose gene-regulatory network in *S. cerevisiae*. The permease Gal2p facilitates intracellular galactose transport. Galactose activates the signal transducer Gal3p, which then sequesters the transcriptional repressor Gal80p. Repression of Gal80p liberates the transcriptional activator Gal4p to up-regulate the GAL genes. The enzymatic pathway transforms galactose into glucose-6-phosphate for glycolysis through the activities of the galactokinase Gal1p, transferase Gal7p, and epimerase Gal10p. The regulatory proteins Gal2p, Gal3p, and Gal80p form positive, positive, and negative feedback loops, respectively. Gal1p, a paralogue of Gal3p, also functions as a signal transducer by interacting with galactose and sequestering Gal80p leading to GAL gene activation [14]. Glucose is imported into the cell by a set of different hexose transporters (HXTi). Inside the cell, glucose activates a regulatory cascade to repress GAL gene expression that includes a set of transcriptional repressors such as Mig1p, Mig2p, Nrg1p, and Nrg2p. These DNA-binding repressors recruit the Cyc8-Tup1 general corepression complex to down-regulate gene expression.
(TIFF)

S2 Fig Distinct gene-expression behaviors arise in response to simultaneous glucose-galactose stimulation despite identical growth conditions for all cultures. Cultures were grown in a microtiter plate at low cell density and diluted with fresh media for 3–6 h prior to induction with the mixed sugar input. **(A)** Cells per microliter (cells/μl) at each time point before induction with a step input of glucose and galactose (top row). Numbers above the top row denote the percentage of glucose added to each culture. All conditions were induced with 0.25% galactose. Bottom row: Single-cell fluorescence distributions of pGAL10-Venus over time obtained using automated flow cytometry for the cultures shown in top row (representative data from Fig. 1). **(B)** Cells per microliter (top) of cultures grown with a different protocol including exponential phase for 24 h then dilution every 20 min for 6 h with fresh media prior to induction with a pulse of glucose and galactose. Fluorescence of pGAL10-Venus for 21.6 h (bottom) following induction with 0.25% galactose and 0.13%, 0.5%, or 2% glucose. Data for panel **A** can be found in S1 Data and data for **B** can be found in S1 Data and S2 Data.
(TIFF)

S3 Fig Flow cytometry distributions as a function of time of a fluorescent protein (Venus) fusion to the *GAL1*, *GAL2*, *GAL7*, and *GAL10* promoters in response to 0.1% glucose and 0.1% galactose. The dashed line denotes the threshold for computing the fraction of ON cells in Fig. 2. The data for this figure can be found in S2 Data.
(TIFF)

S4 Fig Fraction of ON cells (F_ON_, see Materials and Methods) as a function of time for a mixture of glucose and galactose. Cells were exposed to the two sugars simultaneously at the beginning of the experiment. **(A)** F_ON_ as a function of time in response to 0.25% glucose and a range of galactose concentrations. Highlighted box in **A** and **B** indicates bimodal region. **(B)** Rate of change of F_ON_ as a function of time for data shown in **A**. A 5-point moving average was applied to the data. **(C)** F_ON_ as a function of time in response to 0.25% galactose and a range of glucose concentrations. Data for panels **A**, **B**, and **C** can be found in S1 Data.
(TIFF)

S5 Fig Flow cytometry time series measurements of sorted ON or OFF subpopulations induced with 1% galactose and 0.25% glucose and then transferred to one of three environments: glucose alone (0.25%), galactose alone (1%), or mixture of glucose and galactose (0.25% glucose and 1% galactose). **(A)** Time evolution of flow cytometry distributions of sorted OFF cells transferred into glucose, galactose, and mixture conditions as described above. **(B)** Time evolution of flow cytometry distributions of sorted ON cells transferred into glucose, galactose, or mixture conditions as described above.
(TIFF)

S6 Fig Fluorescence time-lapse microscopy in ONIX microfluidic devices (CellASIC) of a bimodal population of cells induced with 1% galactose and 0.25% glucose in minimal media for 4 h and then transferred to a microfluidic device with a continuous inflow of media. **(A)** Representative ON and OFF colonies over time. In the microfluidic devices, cells were grown in 1% galactose and 0.25% glucose for 3 h and then switched to 1% and 0.1% glucose for 3 h. Numbers indicate when the image was taken in hours. **(B)** Single-cell fluorescence of two ON (top) and two OFF (bottom) colonies over time. Single-cell fluorescence was computed as described in the Materials and Methods. The inset in the lower-left panel highlights three cells with fluorescence values in the upper tail of the OFF fluorescence distribution (magenta, green, and cyan lines). **(C)** Quantification of the colony growth rates for the ON and OFF subpopulations. Total number of cells over time (top left) for eight ON or OFF colonies. Growth rate of these colonies over time (bottom left). A 5-point moving average was applied to the data. Mean growth rate over time (right). The shaded regions represent one s.d. from the mean (*n* = 8). From 3–6 h, mean growth rates between the ON and OFF colonies are statistically different with a *p*-value of 8.8e-04. Data for panels **B** and **C** can be found in S1 Data.
(TIFF)

S7 Fig Experimentally measured dynamic features of the GAL system as a function of glucose and galactose. (**A**) Duration of bimodality (δ_g_) as a function of galactose for different glucose concentrations. **(B)** Representative means of the ON and OFF subpopulations over time quantified using a Gaussian mixture model (GMM) for 0.063% glucose and 0.25% galactose for a range of initial population sizes (N_o_). Each marker represents a culture that was initialized with a different number of cells (arrow highlights that increasing N_o_ decreases the duration of bimodality—δ_g_). **(C)** The relationship between the values of N_o_ and δ_g_ for three concentrations of glucose. **(D)** Two steps of glucose produce a larger δ_g_. Representative means of the OFF and ON subpopulations as a function of time for cultures that either received a single step of 0.063% glucose (red circles) or two steps of 0.063% glucose (one at time zero and the second after 5 h, blue squares). Both cultures also received 0.25% galactose at time zero. **(E)** Quantification of δg across a range of N_o_ for three glucose concentrations in conditions with a single or two steps of glucose. All conditions received 0.25% galactose from time zero. Data point size is proportional to N_o_. **(F)** Comparison of the fraction of ON cells at the midpoint of the transient bimodal region (F_ON-mid_, see Materials and Methods) across a range of N_o_ for three glucose concentrations for conditions that received one or two steps of glucose. All conditions also received 0.25% galactose from time zero. Data point size is proportional to N_o_. Data for panels **A**–**D** and **F** can be found in S1 Data.
(TIFF)

S8 Fig Modulation of the duration of bimodality (δ_g_) and fraction of ON cells at the midpoint of the transient bimodal region (F_ON-mid_, see Materials and Methods) in a panel of mutants. **(A)** Means of the ON and OFF subpopulations for a set of mutants that exhibited a larger δ_g_ compared to wild type. These mutants include gene deletions of *CTI6* (A-1) and *HXK2* (A-2) in response to a step input of 0.05% glucose and 0.13% galactose. Disruption of the feedback loop of *GAL80* by a deletion of this gene and expression of *GAL80* from an inducible aTc-responsive promoter (19.7 ng/ml aTc, GAL80Δ fb) prevents the OFF subpopulation from activating for the duration of the experiment in the presence of 0.06% glucose and 0.5% galactose (A-3). **(B)** Means of the ON and transiently OFF subpopulations for a set of mutants that exhibited a smaller δ_g_ compared to WT. These mutants include a quadruple point mutant of Mig1p (Mig1_4m_) that reduces the affinity of Mig1p to the general repression complex Cyc8-Tup1 (B-1) in response to 0.05% glucose and 0.13% galactose, hemizygous mutant of *CYC8* and *TUP1* in a diploid strain compared to a wild-type diploid (B-2) for a step input of 0.05% glucose and 0.13% galactose and single point mutants of Gal4p (F856C and M861C) with reduced affinity to Gal80p (B-3) for a step input of 0.08% glucose and 0.13% galactose. **(C)** Quantification of δ_g_ in response to a step of 0.13% galactose and a range of glucose levels. δ_g_ could not be quantified for mutants in **A** that exhibited an OFF subpopulation that never switched to the ON state over the course of the experiment. **(D)** F_ON-mid_ for the same mutants and doses of glucose and galactose as panel **C**. **(E)** Growth rates of the mutant strains. Growth rates were computed by linear regression on the number of cells collected at each time point averaged over a period of 12 h. Data for panels **A** can be found in S1 Data.
(TIFF)

S9 Fig Populations of cells from different growth stages (exponential or stationary phase) exhibited transient bimodality as a function of glucose and galactose. **(A)** Single cell fluorescence distributions of pGAL10-Venus as a function of time for wild type haploid *S. cerevisiae* W303 cultures with an exponential phase history obtained using automated flow-cytometry. Cultures were given a mixture of 1% galactose and a range of glucose concentrations (from left to right: 0.14%, 0.2%, 0.3%, 0.4%, 0.7%, and 1%). The sugar stimulus was provided initially to cells at the beginning of the experiment and YP media lacking the sugar was added at every subsequent time point thus producing a pulse (see Materials and Methods). In each subplot, the *x*-axis is time and the *y*-axis is fluorescence. **(B)** Single cell fluorescence distributions of pGAL10-Venus as a function of time in wild type *S. cerevisiae* cultures with a stationary history obtained using automated flow-cytometry. Sugar conditions and axes are as in **A**. **(C)** Quantification of F_ON_ over time of the fluorescence distributions in **A** and **B**. **(D)** F_ON-mid_ as a function of glucose for data in **A** and **B**. **(E)** δ_g_ as a function of glucose for data in **A** and **B**. **(F)** δ_a_ as a function of glucose for data in **A** and **B**. Data for panels **C, D, E**, and **F** can be found in S1 Data.
(TIFF)

S10 Fig The transient bimodal response to mixtures of sugars is preserved for different carbon source histories (YP, YP + 3% glycerol or YP + 3% ethanol). **(A)** Single cell fluorescence distributions of pGAL10-Venus as a function of time in wild type haploid *S. cerevisiae* W303 obtained using automated flow-cytometry. In each subplot, the *x*-axis is time and the *y*-axis is fluorescence. Cell populations were grown in YP (top), YP + 3% glycerol (middle) or YP + 3% ethanol (bottom). Cultures were given a mixture of 1% galactose and a range of glucose concentrations (from left to right: 0.14%, 0.2%, 0.3%, 0.4%, 0.7%, and 1%). The sugar stimulus was provided to cells at the beginning of the experiment and YP media lacking the sugar was added at every subsequent time point thus producing a pulse (see Materials and Methods). **(B)** Quantification in terms of F_ON_ over time of distributions in **A**. **(C)** F_ON-mid_ as a function of glucose for data in **A**. **(D)** δ_g_ as a function of glucose for data in **A**. **(E)** δ_a_ as a function of glucose for data in **A**. Data for panels **A**, **C, D**, and **E** can be found in S1 Data.
(TIFF)

S11 Fig Comparison of sugar consumption for WT *S. cerevisiae* containing a pGAL10-Venus reporter that received 0.1% galactose or mixture of 0.1% galactose + 0.1% glucose. **(A)** Galactose consumption (blue) and F_ON_ (green) for cells that received 0.1% galactose. **(B)** Galactose (blue), glucose (green) and F_ON_ (red) over time for cells that received 0.1% glucose + 0.1% galactose. **(C)** Comparison between the rate at which the ON subpopulation consumes galactose over time for the cell population that received only galactose (red), glucose and galactose mixture (green) and the expected consumption rate based on the galactose only condition (blue, see Materials and Methods). **(D)** Relationship between galactose and fluorescence output for the Amplex Red Galactose kit (Life Technologies). The high galactose measurements are in the linear range as determined by a galactose standard. **(E)** Relationship between glucose and fluorescence output for the Amplex Red Glucose kit (Life Technologies). The high glucose measurements are in the linear regime based on the glucose standard. Data for panels **A**–**E** can be found in S1 Data.
(TIFF)

S12 Fig The protein levels of the Gal2 permease strongly correlate with a *GAL10* promoter fusion in response to a mixture of glucose and galactose. **(A)** Flow cytometry distributions of a single strain expressing both a fluorescent protein fusion of Venus to Gal2p (pGAL2-GAL2-Venus) and a promoter fusion of mCherry to the *GAL10* promoter (pGAL10-mCherry) following a 5 h induction with different combinations of glucose and galactose. **(B)** Heat-map of correlation coefficients of pGAL2-GAL2-Venus and pGAL10-mCherry for the distributions shown in **A**. **(C)** Microscopy images of wild-type cells expressing pGAL2-GAL2-Venus exposed to either 1% galactose (top) or 1% galactose + 0.25% glucose (bottom) for 4 h. **(D)** Relationship between the Gal2p expression level and the fraction of ON cells (F_ON_) in a strain deleted for the endogenous *GAL2* gene and expressing Gal2p from an aTc inducible TET promoter and pGAL10-Venus reporter (GAL2Δ fb). Cells were exposed to 0.5% galactose and a range of glucose concentrations and aTc levels (ng/μl) for 6 h. Data for panels **B** and **D** can be found in S1 Data.
(TIFF)

S13 Fig A possible model where the effect of glucose on the GAL network is indirect through modulation of the cellular growth rate which modifies the linear decay rates of the GAL proteins. **(A)** GAL network diagram of the signal transducer Gal1p (G1), repressor Gal80p (G80) and transcription activator Gal4p (G4). In this model, galactose activates the G1 to form G1*, which sequesters G80 from inhibiting G4 by sequestration. The glucose input increases the linear decay rate (dilution rate) of all species in the model by a different scaling factor (see S1 Text). **(B)** Bifurcation diagram at steady-state. The bifurcation parameters were the degradation rate parameters and galactose. Random parameter sampling (see S1 Text) within physiologically realistic bounds identified a parameter set in which varying the degradation rates in the model can trigger bistability. MATLAB code for panel **B** can be found in S2 Data and data for panel **B** can be found in S1 Data.
(TIFF)

S14 Fig Initialization of the GAL model with glucose inhibition in different concentration regimes of glucose and galactose generates distinct dynamic responses. The beginning of the experiment is denoted by t_0_. The solid line illustrates the sequential consumption of glucose and galactose as a function of time. The colored regions denote monostability and the white region represents bistability. In the colored regions, red indicates monostable ON corresponding to high total G1 levels (G1_T_) and blue represents monostable OFF corresponding to low G1_T_. **(A)** Representative dynamic trajectory in response to galactose and a sufficiently low initial glucose concentration. **(B)** Representative dynamic trajectory in response to an initial glucose concentration that is significantly higher than the initial galactose level. **(C)** Representative dynamic trajectory in response to a mixture of glucose and galactose that produces bistability in the model for the parameter set listed in S1 Table. **(D)** Representative dynamic trajectory in response to the same concentrations of glucose and galactose as (C) for a 20% reduction in the affinity of the glucose-dependent repressor R* to the *G1* and *G4* promoters (parameter set listed in S1 Table, K_R1_ = 80.8 nM and K_R4_ = 40.6 nM).
(TIFF)

S15 Fig Removal of the *GAL80* feedback loop expands the region of bistability across different concentrations of glucose and galactose in the computational model. This feature is corroborated by experimental measurements of a strain in which the *GAL80* gene is deleted and this gene is expressed from an inducible aTc-responsive promoter in response to 0 ng/ml aTc (GAL80Δ fb). This level of constitutive Gal80p expression corresponds to approximately 40% of fully induced wild type levels [14]. **(A)** Circuit diagram of the GAL80Δ fb model (left, see S1 Text). Bifurcation diagram of the regions of monostability (colored) and bistability (white) for different values of glucose and galactose (right). The model predicts that removing the *GAL80* feedback loop expands the range of glucose and galactose concentrations that produce bistability. **(B)** Single-cell fluorescence distributions of pGAL10-Venus in the wild type and GAL80Δ fb. **(C)** Gaussian mixture model (GMM) classification (see Materials and Methods) of experimentally measured pGAL10-Venus gene expression distributions shown in **B** for the wild type. Red and blue represent a bimodal and monomodal distribution, respectively. **(D)** GMM classification of experimentally measured fluorescence distributions in **B** for GAL80Δ fb. Data for panels **C** and **D** can be found in S1 Data.
(TIFF)

S16 Fig Single-cell fluorescence distributions of pGAL10-Venus in wild type for the conditions in which galactose was added at 0, 3.1, 4.2, 5.3, and 6.3 h (Figs. 3 and 4). The dotted lines denote the threshold used to compute the fraction of ON cells in Fig. 3.
(TIFF)

S17 Fig Optical density (OD_600_) and growth rates over time for the delayed galactose experiment (Figs. 3 and 4). **(A)** Optical density over time. Dashed lines indicate approximate diauxic shift. **(B)** Mean doubling rate for each condition during the diauxic shift (dashed lines in **A**). Data for panels **A** and **B** can be found in S1 Data.
(TIFF)

S18 Fig Galactose does not benefit cells until glucose is consumed for a mutant strain in which the *GAL4* gene is deleted and a point mutant of *GAL4* (C14Y) is expressed that is unable to activate GAL gene expression (Gal4 DBD*). Cells were exposed to 0.1% glucose and 0.1% galactose at the beginning of the experiment. **(A)** Single-cell fluorescence distributions of a pGAL10-Venus in the wild type and Gal4 DBD*. **(B)** Glucose concentrations as a function of time. Lines represent fitted Hill functions. **(C)** Galactose concentrations as a function of time. **(D)** Growth rates as a function of time. Data for panels **B**, **C**, and **D** can be found in S1 Data.
(TIFF)

S19 Fig Early activation of the GAL genes using an estradiol inducible Gal4 chimera prior to glucose depletion reduces the rate of glucose consumption and accelerates galactose consumption. **(A)** Experimental design. Each condition (A-E) received a step input of 0.1% glucose and 0.1% galactose at time zero and 500 nM estradiol to activate the Gal4 chimera at the indicated times (top). Single-cell fluorescence distributions of pGAL10-Venus as a function of time (bottom). **(B)** Glucose concentrations as a function of time. Lines represent fitted Hill functions. Dashed lines indicate the time when estradiol was added to each culture. **(C)** Normalized change in the half-max of the glucose consumption curves for the different conditions relative to condition A. **(D)** Galactose concentrations as a function of time. **(E)** Fractional change in the half-max of the galactose concentration curves relative to condition A. Data for panels **B**, **C**, **D**, and **E** can be found in S1 Data.
(TIFF)

S20 Fig Modification of sugar metabolism and growth by constitutive GAL gene expression. The GAL pathway was induced approximately 12 h in advance of the wild type using a strain in which *GAL3* is deleted and this gene is expressed from an inducible aTc-responsive promoter (450 ng/ml aTc, GAL3Δ fb). At time zero, the GAL3Δ fb and wild-type strains received 0.1% glucose and 0.1% galactose. **(A)** Single-cell fluorescence of pGAL10-Venus in wild type and GAL3Δ fb. **(B)** Glucose concentrations as a function of time. Lines represent fitted Hill functions. **(C)** Galactose concentrations as a function of time. **(D)** Relationship between the glucose and galactose concentrations for the WT and GAL3Δ fb strains. **(E)** Metabolic delay for wild type and GAL3Δ fb computed by subtracting the half-max of the glucose and galactose curves. **(F)** Growth rates as a function of time. Data for panels **B**, **C**, **D**, **E**, and **F** can be found in S1 Data.
(TIFF)

S21 Fig The GAL OFF subpopulation grows faster than the GAL ON subpopulation. **(A)** Representative dynamic flow cytometry measurements of pGAL10-Venus in response to 0.5% glucose and 1% galactose from the experiment in Fig. 1. Heat-map of fluorescence distributions as a function of time (left). Quantification of the number of cells in the OFF and ON subpopulations (right, see Materials and Methods). Log_2_N as a function of time. N represents the number of cells in the GAL ON or OFF subpopulations. **(B)** Growth rate of GAL OFF subpopulation versus the GAL ON subpopulation for different initial concentrations of glucose and galactose. The diameter of each data point is proportional to the initial concentration of glucose. Data for panel **B** can be found in S1 Data.
(TIFF)

S22 Fig Fluorescence time-lapse microscopy in ONIX microfluidic devices (CellASIC) of a bimodal population of cells induced with 1% galactose and 0.25% glucose in minimal media containing 2% raffinose for 4 h and then transferred to the microfluidic device with a constant inflow of media. **(A)** Representative ON (top panels) and OFF (bottom panels) colonies over time. In the microfluidic devices, cells were grown in 1% galactose + 0.25% glucose for 3 h and then switched to 1% galactose media. Numbers indicate when the image was taken in hours. **(B)** Quantification of the colony growth rates for the ON and OFF subpopulations. Total number of cells over time (top left) for 13 ON or OFF colonies. Growth rate of these colonies over time (bottom left). Mean growth rate over time (right). The shaded regions represent one s.d. from the mean (*n* = 13). Following the switch to galactose, the difference in the growth rates between the ON and OFF subpopulations is statistically significant with a *p*-value of 9.8e-04. Data for panels **A**, **B**, and **C** can be found in S1 Data. Data for panel **B** can be found in S1 Data.
(TIFF)

S1 Table Parameter values for the ordinary differential equation GAL model with a glucose-dependent repressor. (XLS)

S2 Table Parameter values for the ordinary differential equation GAL model in the absence of a glucose-dependent repressor. (XLS)

S3 Table Strains used in this study. All strains were W303. *These strains have been described previously [14].
(XLSX)

S1 Text Section S1.1 describes the ordinary differential equation model of the galactose regulatory network containing a glucose repressor in Fig. 3. Section S1.2 describes an ordinary differential equation model with glucose-dependent modification of the decay rates corresponding to S13 Fig. Section S2 reports the modification of the bimodality parameters (δ_g_ and F_ON_) in a set of genetic mutants. Section S3 comments on S18 Fig., Section S4 discusses S20 Fig., and Section S5 comments on S21 Fig.
(PDF)

## Abbreviations

δ_g_: delay between early and late activation for conditions with transient bimodality
F_ON_: fraction of ON cells
GAL: galactose gene regulatory network
GMM: Gaussian mixture model
Venus: Yellow Fluorescent Protein
MAL: maltose gene regulatory network
