# Supplementary figures and images for: Population Diversification in a Yeast Metabolic Program Promotes Anticipation of Environmental Shifts
Source: PLoS Biol. 2015 Jan 27;13(1):e1002042. doi: 10.1371/journal.pbio.1002042 (PMC4307983; doi:10.1371/journal.pbio.1002042)

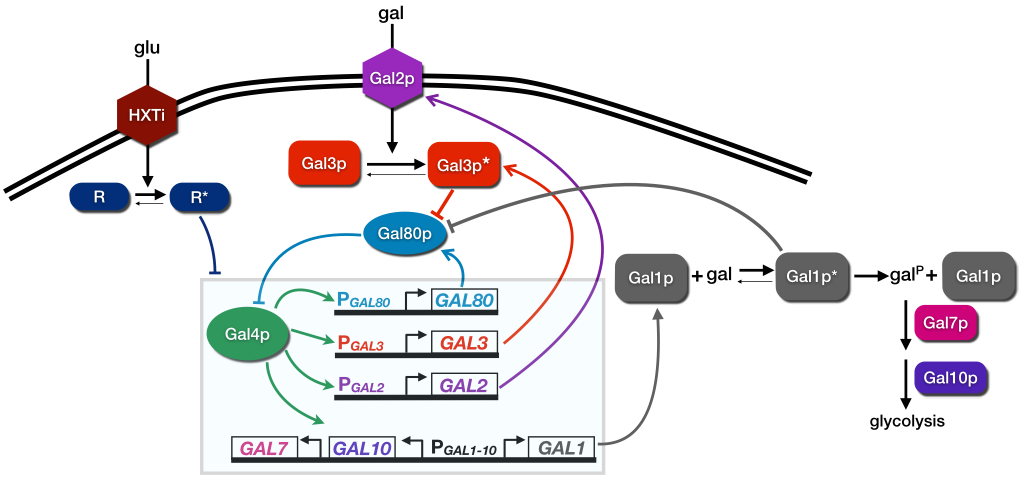

Supplement: S1 Fig — The permease Gal2p facilitates intracellular galactose transport. Galactose activates the signal transducer Gal3p, which then sequesters the transcriptional repressor Gal80p. Repression of Gal80p liberates the transcriptional activator Gal4p to up-regulate the GAL genes. The enzymatic pathway transforms galactose into glucose-6-phosphate for glycolysis through the activities of the galactokinase Gal1p, transferase Gal7p, and epimerase Gal10p. The regulatory proteins Gal2p, Gal3p, and Gal80p form positive, positive, and negative feedback loops, respectively. Gal1p, a paralogue of Gal3p, also functions as a signal transducer by interacting with galactose and sequestering Gal80p leading to GAL gene activation [14]. Glucose is imported into the cell by a set of different hexose transporters (HXTi). Inside the cell, glucose activates a regulatory cascade to repress GAL gene expression that includes a set of transcriptional repressors such as Mig1p, Mig2p, Nrg1p, and Nrg2p. These DNA-binding repressors recruit the Cyc8-Tup1 general corepression complex to down-regulate gene expression. (TIFF) [file pbio.1002042.s003.tiff]

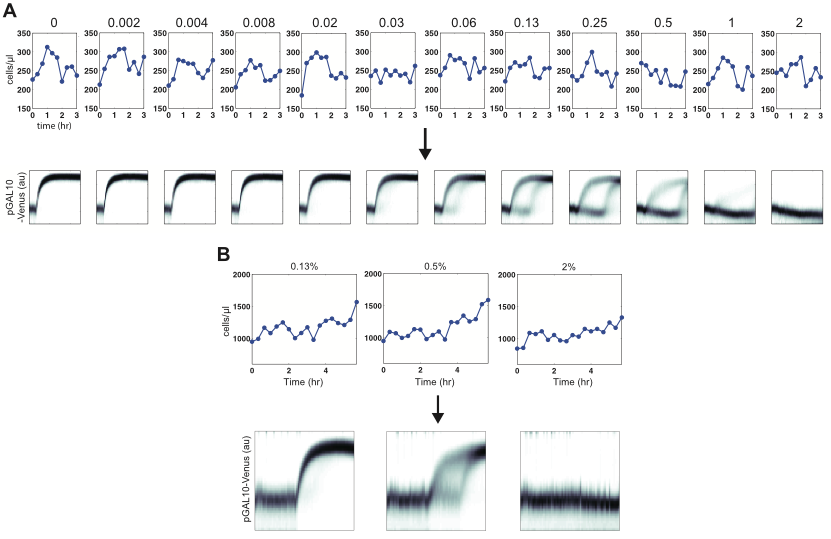

Supplement: S2 Fig — Cultures were grown in a microtiter plate at low cell density and diluted with fresh media for 3–6 h prior to induction with the mixed sugar input. (A) Cells per microliter (cells/μl) at each time point before induction with a step input of glucose and galactose (top row). Numbers above the top row denote the percentage of glucose added to each culture. All conditions were induced with 0.25% galactose. Bottom row: Single-cell fluorescence distributions of pGAL10-Venus over time obtained using automated flow cytometry for the cultures shown in top row (representative data from Fig. 1). (B) Cells per microliter (top) of cultures grown with a different protocol including exponential phase for 24 h then dilution every 20 min for 6 h with fresh media prior to induction with a pulse of glucose and galactose. Fluorescence of pGAL10-Venus for 21.6 h (bottom) following induction with 0.25% galactose and 0.13%, 0.5%, or 2% glucose. Data for panel A can be found in S1 Data and data for B can be found in S1 Data and S2 Data. (TIFF) [file pbio.1002042.s004.tiff]

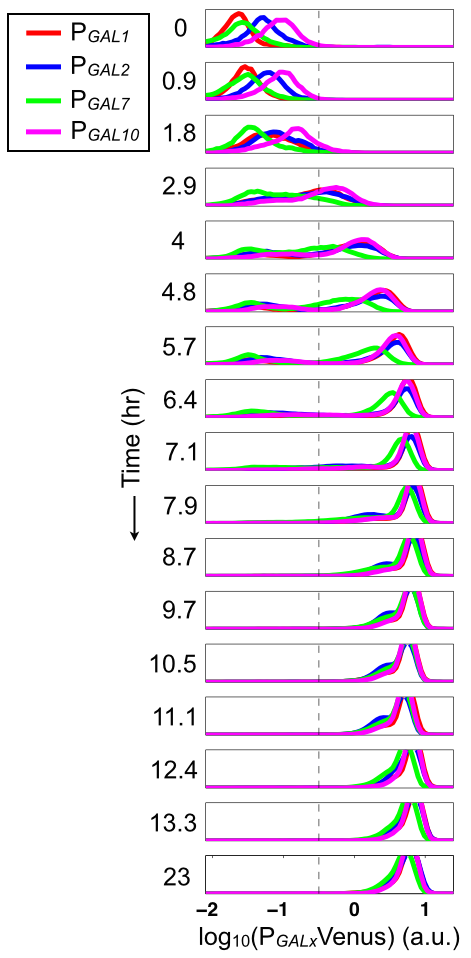

Supplement: S3 Fig — The dashed line denotes the threshold for computing the fraction of ON cells in Fig. 2. The data for this figure can be found in S2 Data. (TIFF) [file pbio.1002042.s005.tiff]

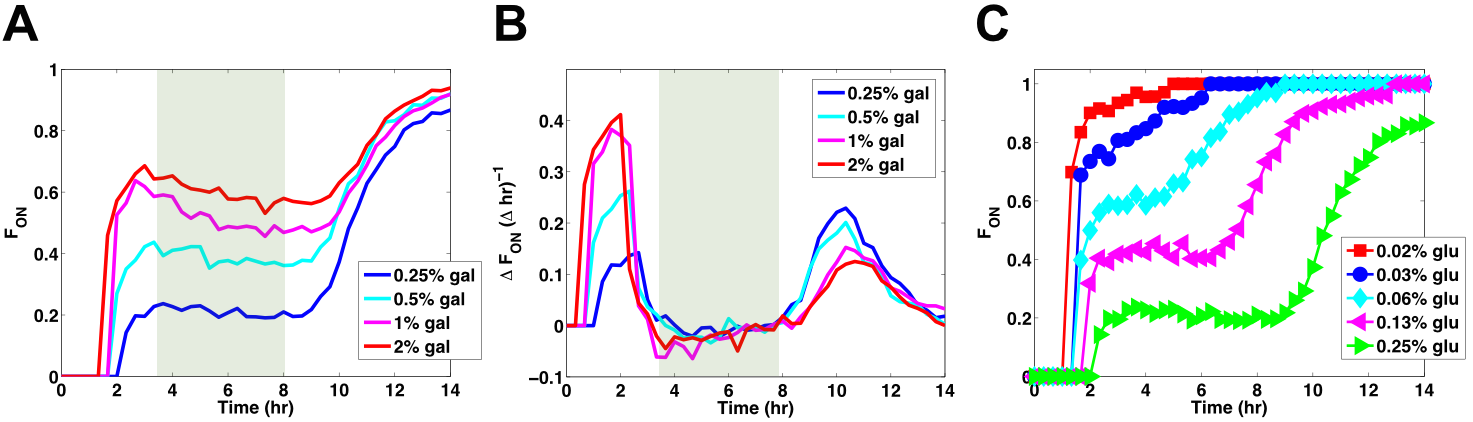

Supplement: S4 Fig — Cells were exposed to the two sugars simultaneously at the beginning of the experiment. (A) FON as a function of time in response to 0.25% glucose and a range of galactose concentrations. Highlighted box in A and B indicates bimodal region. (B) Rate of change of FON as a function of time for data shown in A. A 5-point moving average was applied to the data. (C) FON as a function of time in response to 0.25% galactose and a range of glucose concentrations. Data for panels A, B, and C can be found in S1 Data. (TIFF) [file pbio.1002042.s006.tiff]

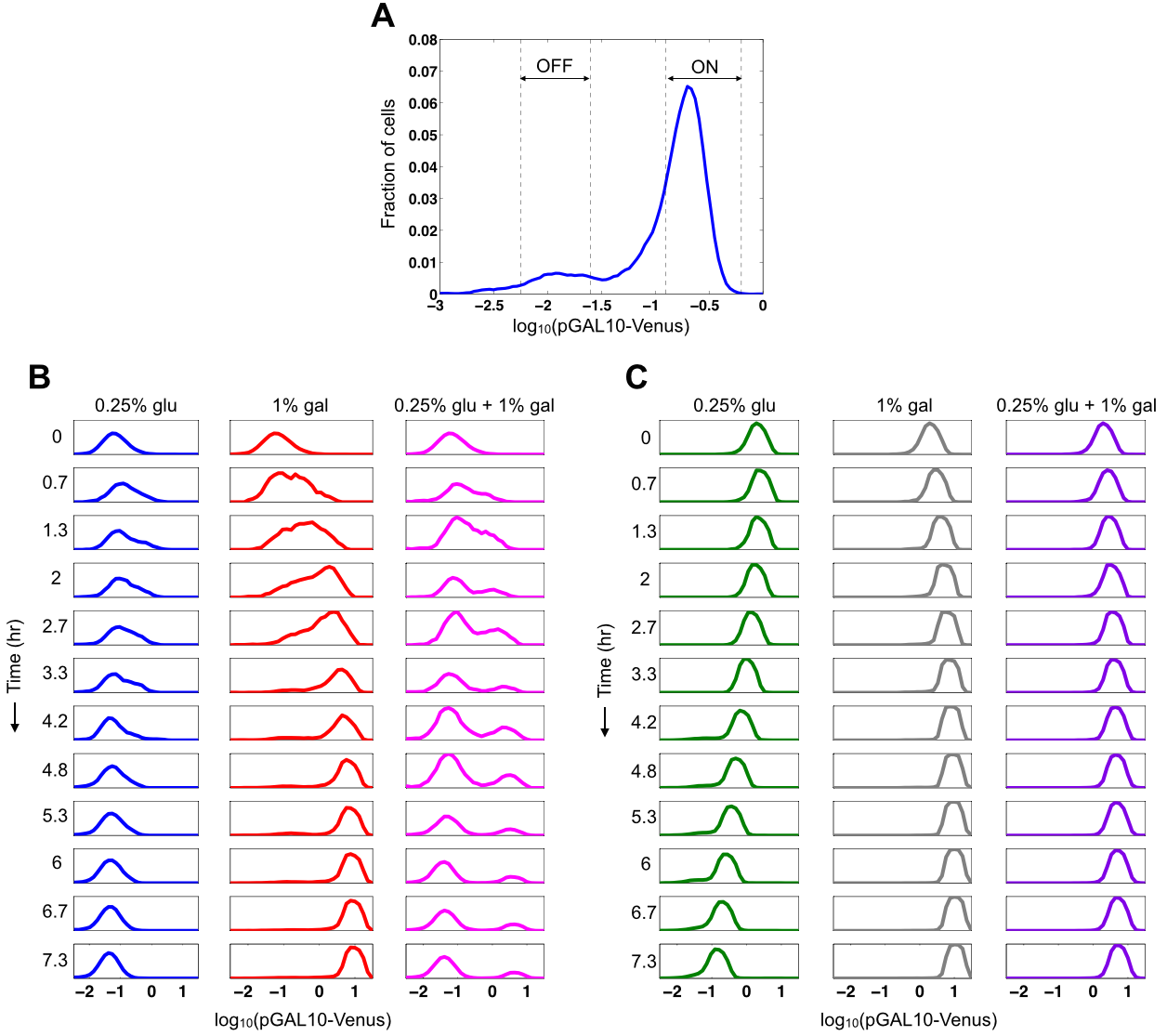

Supplement: S5 Fig — (A) Time evolution of flow cytometry distributions of sorted OFF cells transferred into glucose, galactose, and mixture conditions as described above. (B) Time evolution of flow cytometry distributions of sorted ON cells transferred into glucose, galactose, or mixture conditions as described above. (TIFF) [file pbio.1002042.s007.tiff]

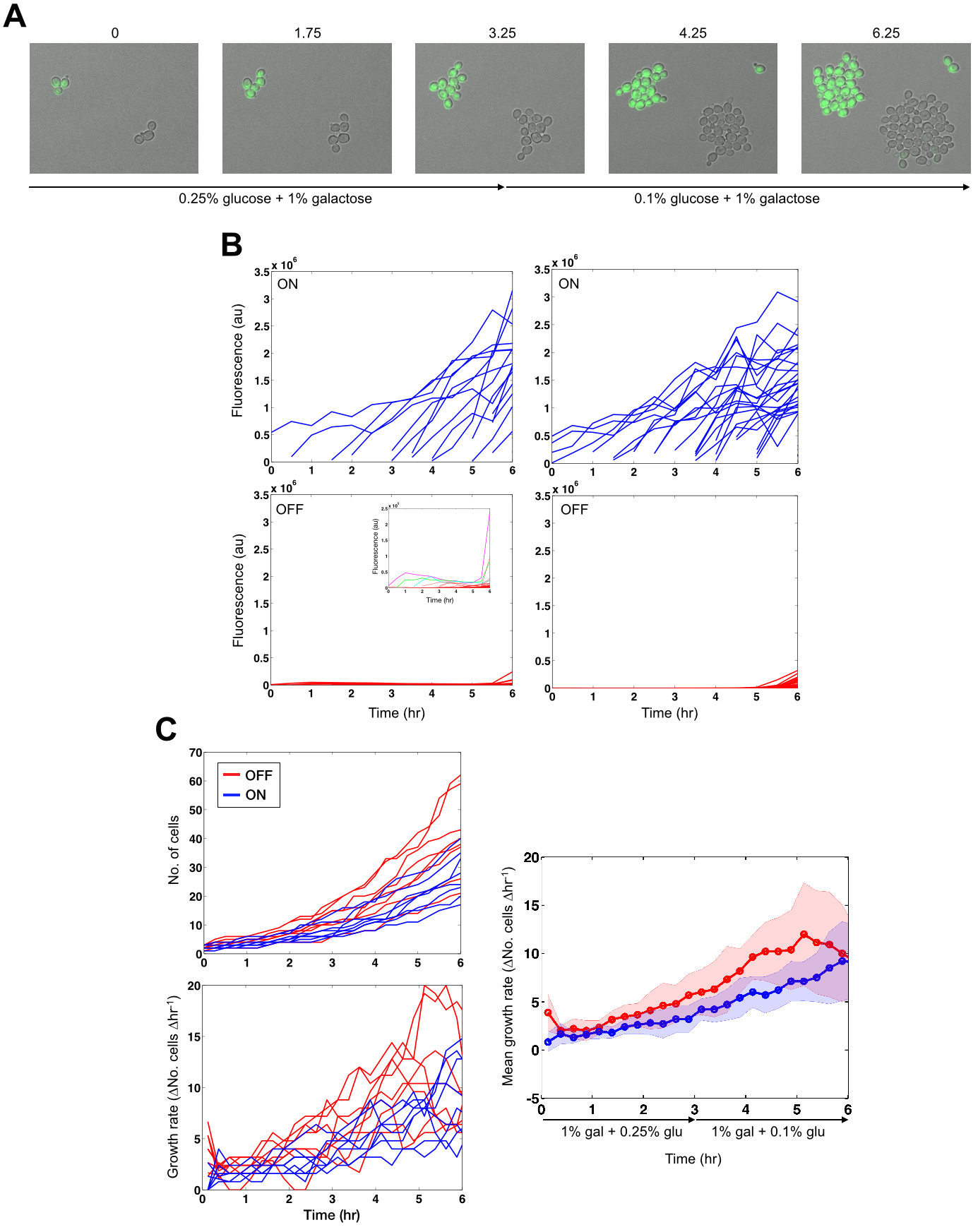

Supplement: S6 Fig — (A) Representative ON and OFF colonies over time. In the microfluidic devices, cells were grown in 1% galactose and 0.25% glucose for 3 h and then switched to 1% and 0.1% glucose for 3 h. Numbers indicate when the image was taken in hours. (B) Single-cell fluorescence of two ON (top) and two OFF (bottom) colonies over time. Single-cell fluorescence was computed as described in the Materials and Methods. The inset in the lower-left panel highlights three cells with fluorescence values in the upper tail of the OFF fluorescence distribution (magenta, green, and cyan lines). (C) Quantification of the colony growth rates for the ON and OFF subpopulations. Total number of cells over time (top left) for eight ON or OFF colonies. Growth rate of these colonies over time (bottom left). A 5-point moving average was applied to the data. Mean growth rate over time (right). The shaded regions represent one s.d. from the mean (n = 8). From 3–6 h, mean growth rates between the ON and OFF colonies are statistically different with a p-value of 8.8e-04. Data for panels B and C can be found in S1 Data. (TIFF) [file pbio.1002042.s008.tiff]

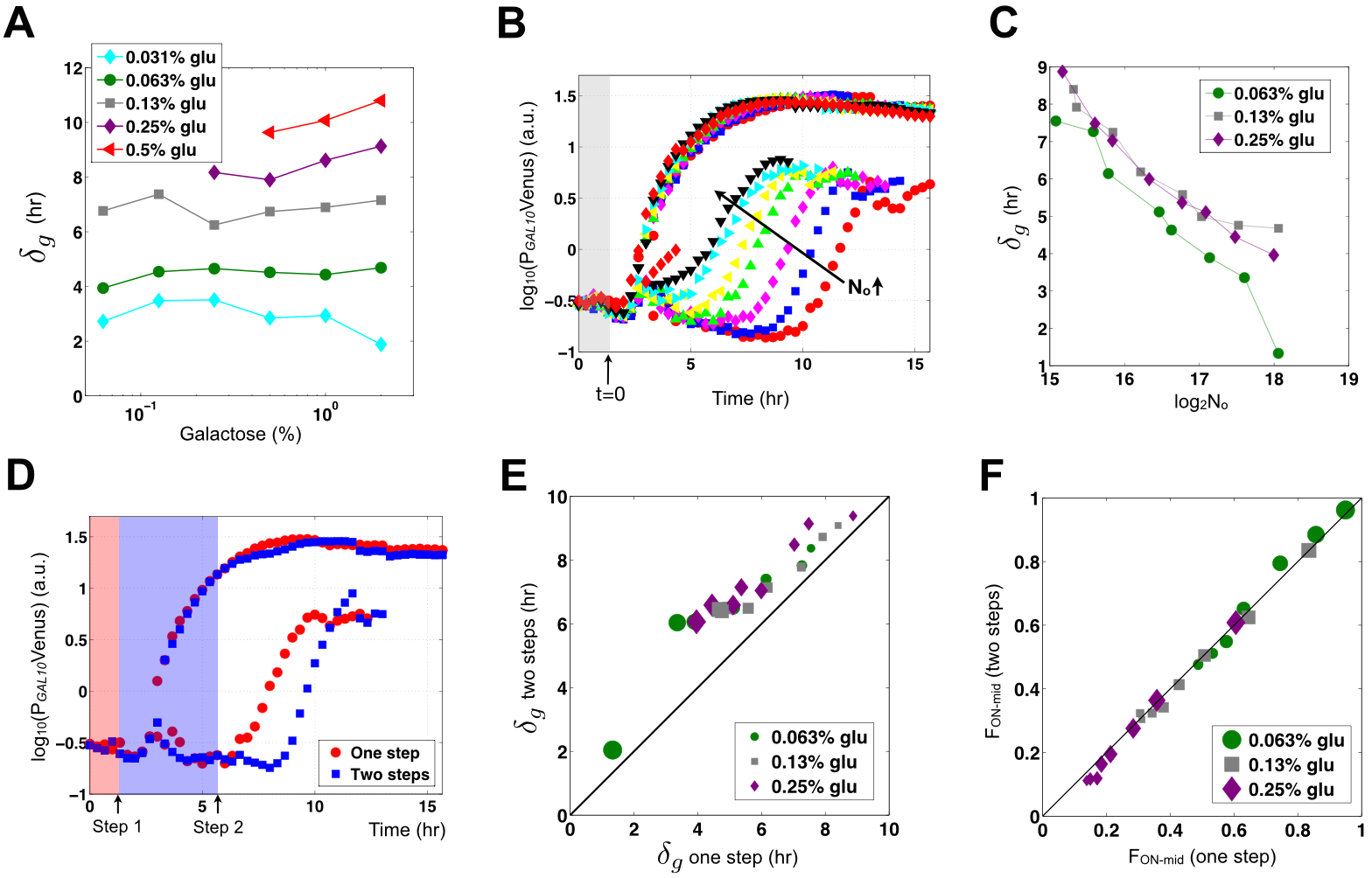

Supplement: S7 Fig — (A) Duration of bimodality (δg) as a function of galactose for different glucose concentrations. (B) Representative means of the ON and OFF subpopulations over time quantified using a Gaussian mixture model (GMM) for 0.063% glucose and 0.25% galactose for a range of initial population sizes (No). Each marker represents a culture that was initialized with a different number of cells (arrow highlights that increasing No decreases the duration of bimodality—δg). (C) The relationship between the values of No and δg for three concentrations of glucose. (D) Two steps of glucose produce a larger δg. Representative means of the OFF and ON subpopulations as a function of time for cultures that either received a single step of 0.063% glucose (red circles) or two steps of 0.063% glucose (one at time zero and the second after 5 h, blue squares). Both cultures also received 0.25% galactose at time zero. (E) Quantification of δg across a range of No for three glucose concentrations in conditions with a single or two steps of glucose. All conditions received 0.25% galactose from time zero. Data point size is proportional to No. (F) Comparison of the fraction of ON cells at the midpoint of the transient bimodal region (FON-mid, see Materials and Methods) across a range of No for three glucose concentrations for conditions that received one or two steps of glucose. All conditions also received 0.25% galactose from time zero. Data point size is proportional to No. Data for panels A–D and F can be found in S1 Data. (TIFF) [file pbio.1002042.s009.tiff]

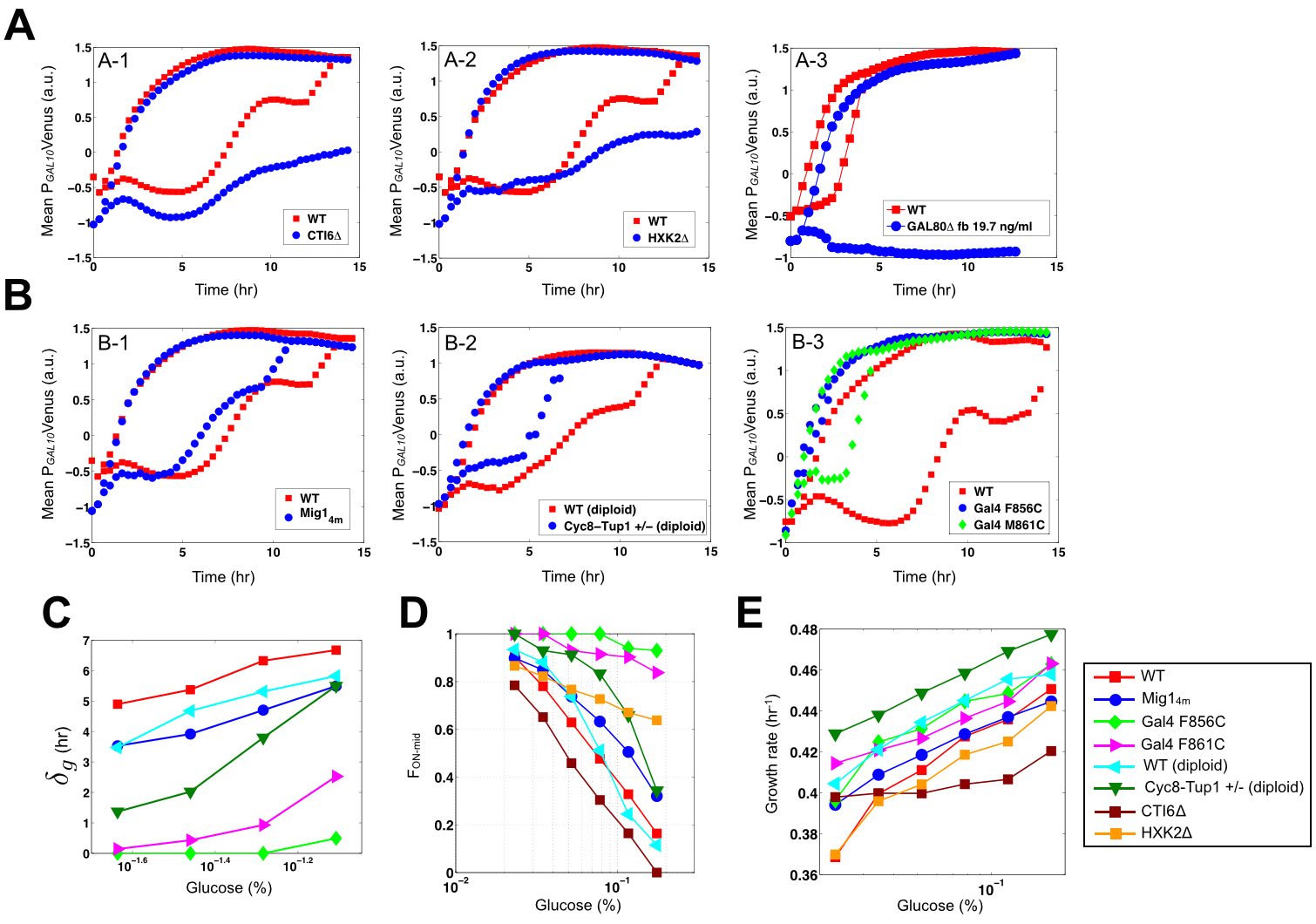

Supplement: S8 Fig — (A) Means of the ON and OFF subpopulations for a set of mutants that exhibited a larger δg compared to wild type. These mutants include gene deletions of CTI6 (A-1) and HXK2 (A-2) in response to a step input of 0.05% glucose and 0.13% galactose. Disruption of the feedback loop of GAL80 by a deletion of this gene and expression of GAL80 from an inducible aTc-responsive promoter (19.7 ng/ml aTc, GAL80Δ fb) prevents the OFF subpopulation from activating for the duration of the experiment in the presence of 0.06% glucose and 0.5% galactose (A-3). (B) Means of the ON and transiently OFF subpopulations for a set of mutants that exhibited a smaller δg compared to WT. These mutants include a quadruple point mutant of Mig1p (Mig14m) that reduces the affinity of Mig1p to the general repression complex Cyc8-Tup1 (B-1) in response to 0.05% glucose and 0.13% galactose, hemizygous mutant of CYC8 and TUP1 in a diploid strain compared to a wild-type diploid (B-2) for a step input of 0.05% glucose and 0.13% galactose and single point mutants of Gal4p (F856C and M861C) with reduced affinity to Gal80p (B-3) for a step input of 0.08% glucose and 0.13% galactose. (C) Quantification of δg in response to a step of 0.13% galactose and a range of glucose levels. δg could not be quantified for mutants in A that exhibited an OFF subpopulation that never switched to the ON state over the course of the experiment. (D) FON-mid for the same mutants and doses of glucose and galactose as panel C. (E) Growth rates of the mutant strains. Growth rates were computed by linear regression on the number of cells collected at each time point averaged over a period of 12 h. Data for panels A can be found in S1 Data. (TIFF) [file pbio.1002042.s010.tiff]

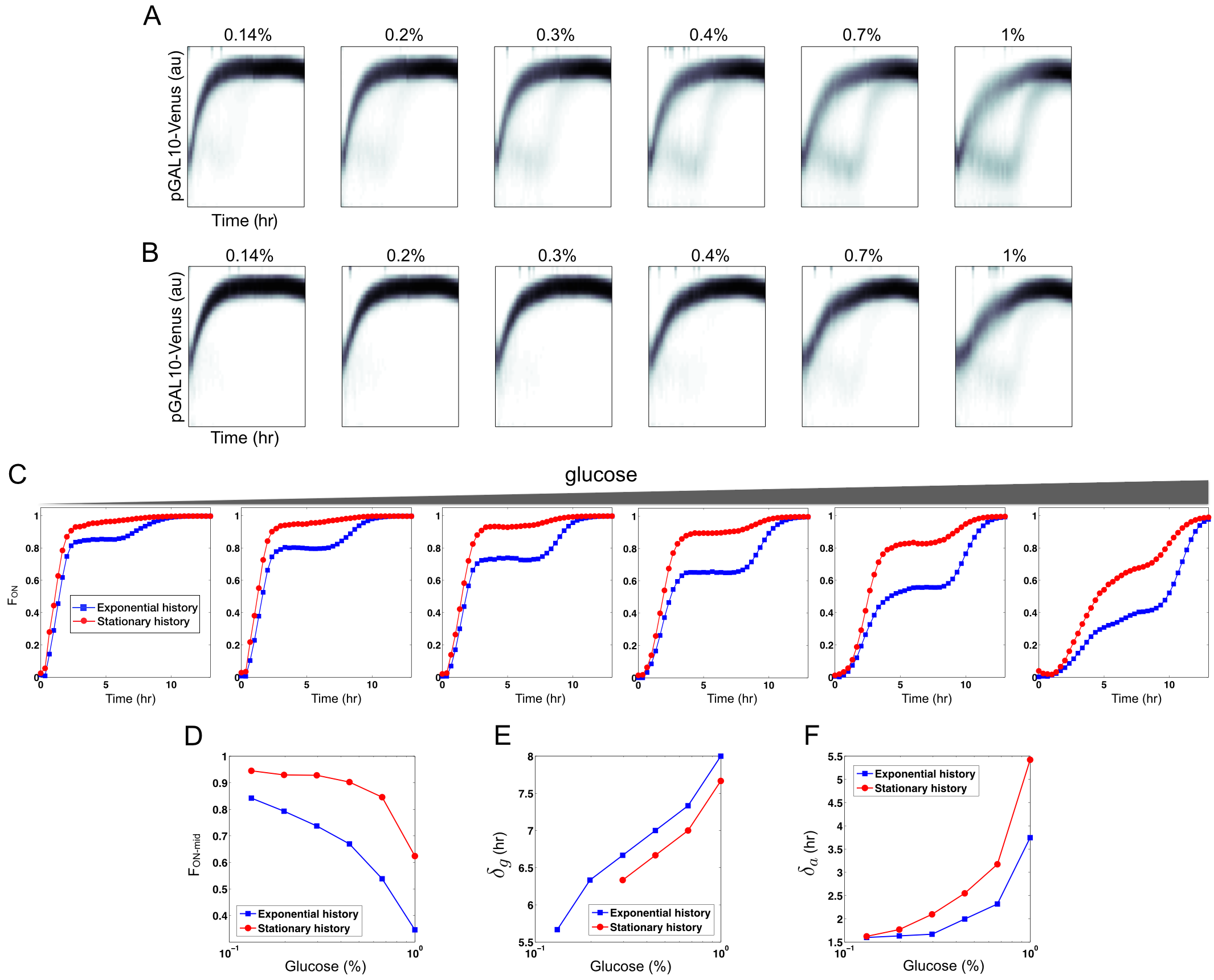

Supplement: S9 Fig — (A) Single cell fluorescence distributions of pGAL10-Venus as a function of time for wild type haploid S. cerevisiae W303 cultures with an exponential phase history obtained using automated flow-cytometry. Cultures were given a mixture of 1% galactose and a range of glucose concentrations (from left to right: 0.14%, 0.2%, 0.3%, 0.4%, 0.7%, and 1%). The sugar stimulus was provided initially to cells at the beginning of the experiment and YP media lacking the sugar was added at every subsequent time point thus producing a pulse (see Materials and Methods). In each subplot, the x-axis is time and the y-axis is fluorescence. (B) Single cell fluorescence distributions of pGAL10-Venus as a function of time in wild type S. cerevisiae cultures with a stationary history obtained using automated flow-cytometry. Sugar conditions and axes are as in A. (C) Quantification of FON over time of the fluorescence distributions in A and B. (D) FON-mid as a function of glucose for data in A and B. (E) δg as a function of glucose for data in A and B. (F) δa as a function of glucose for data in A and B. Data for panels C, D, E, and F can be found in S1 Data. (TIFF) [file pbio.1002042.s011.tiff]

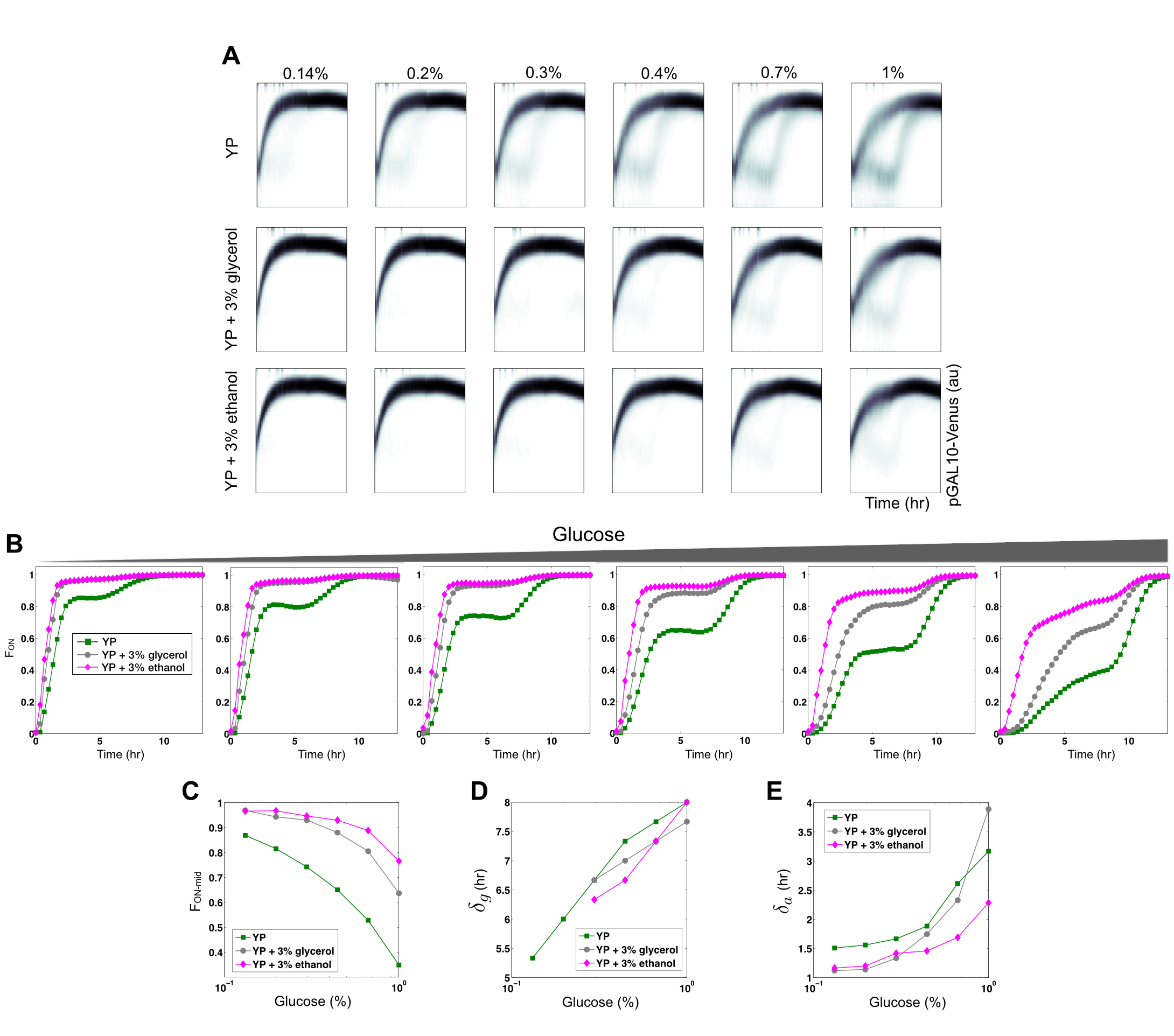

Supplement: S10 Fig — (A) Single cell fluorescence distributions of pGAL10-Venus as a function of time in wild type haploid S. cerevisiae W303 obtained using automated flow-cytometry. In each subplot, the x-axis is time and the y-axis is fluorescence. Cell populations were grown in YP (top), YP + 3% glycerol (middle) or YP + 3% ethanol (bottom). Cultures were given a mixture of 1% galactose and a range of glucose concentrations (from left to right: 0.14%, 0.2%, 0.3%, 0.4%, 0.7%, and 1%). The sugar stimulus was provided to cells at the beginning of the experiment and YP media lacking the sugar was added at every subsequent time point thus producing a pulse (see Materials and Methods). (B) Quantification in terms of FON over time of distributions in A. (C) FON-mid as a function of glucose for data in A. (D) δg as a function of glucose for data in A. (E) δa as a function of glucose for data in A. Data for panels A, C, D, and E can be found in S1 Data. (TIFF) [file pbio.1002042.s012.tif]

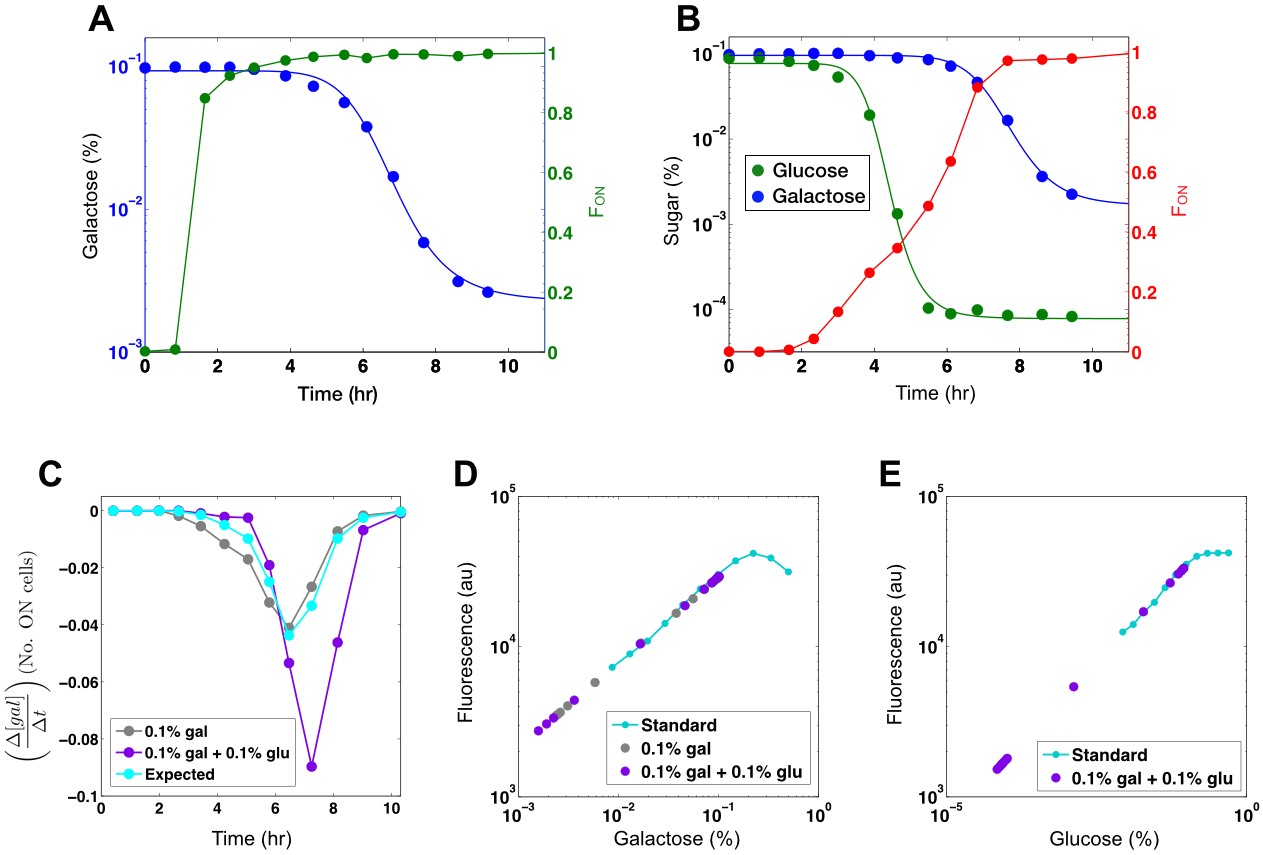

Supplement: S11 Fig — (A) Galactose consumption (blue) and FON (green) for cells that received 0.1% galactose. (B) Galactose (blue), glucose (green) and FON (red) over time for cells that received 0.1% glucose + 0.1% galactose. (C) Comparison between the rate at which the ON subpopulation consumes galactose over time for the cell population that received only galactose (red), glucose and galactose mixture (green) and the expected consumption rate based on the galactose only condition (blue, see Materials and Methods). (D) Relationship between galactose and fluorescence output for the Amplex Red Galactose kit (Life Technologies). The high galactose measurements are in the linear range as determined by a galactose standard. (E) Relationship between glucose and fluorescence output for the Amplex Red Glucose kit (Life Technologies). The high glucose measurements are in the linear regime based on the glucose standard. Data for panels A–E can be found in S1 Data. (TIFF) [file pbio.1002042.s013.tiff]

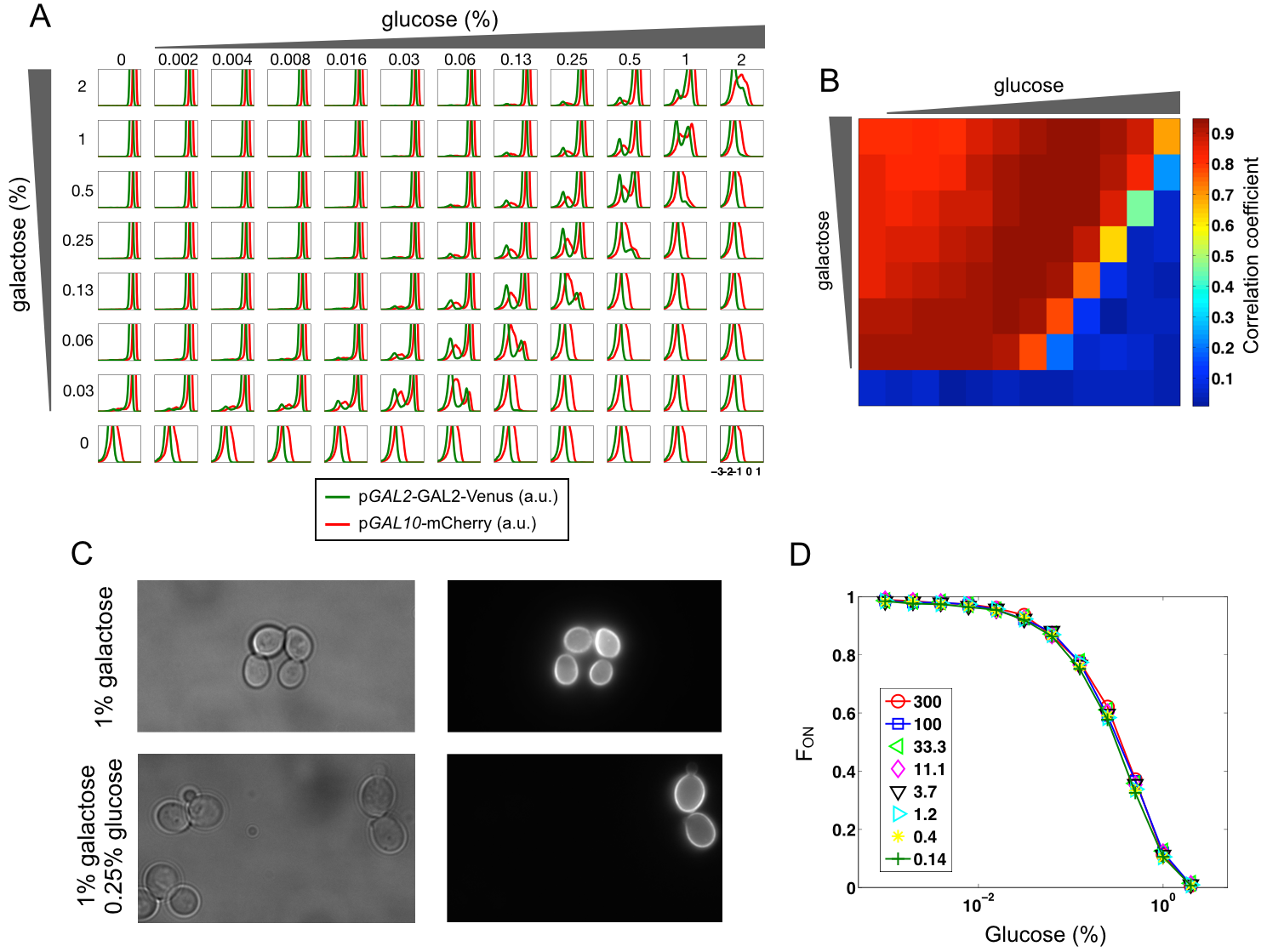

Supplement: S12 Fig — (A) Flow cytometry distributions of a single strain expressing both a fluorescent protein fusion of Venus to Gal2p (pGAL2-GAL2-Venus) and a promoter fusion of mCherry to the GAL10 promoter (pGAL10-mCherry) following a 5 h induction with different combinations of glucose and galactose. (B) Heat-map of correlation coefficients of pGAL2-GAL2-Venus and pGAL10-mCherry for the distributions shown in A. (C) Microscopy images of wild-type cells expressing pGAL2-GAL2-Venus exposed to either 1% galactose (top) or 1% galactose + 0.25% glucose (bottom) for 4 h. (D) Relationship between the Gal2p expression level and the fraction of ON cells (FON) in a strain deleted for the endogenous GAL2 gene and expressing Gal2p from an aTc inducible TET promoter and pGAL10-Venus reporter (GAL2Δ fb). Cells were exposed to 0.5% galactose and a range of glucose concentrations and aTc levels (ng/μl) for 6 h. Data for panels B and D can be found in S1 Data. (TIFF) [file pbio.1002042.s014.tiff]

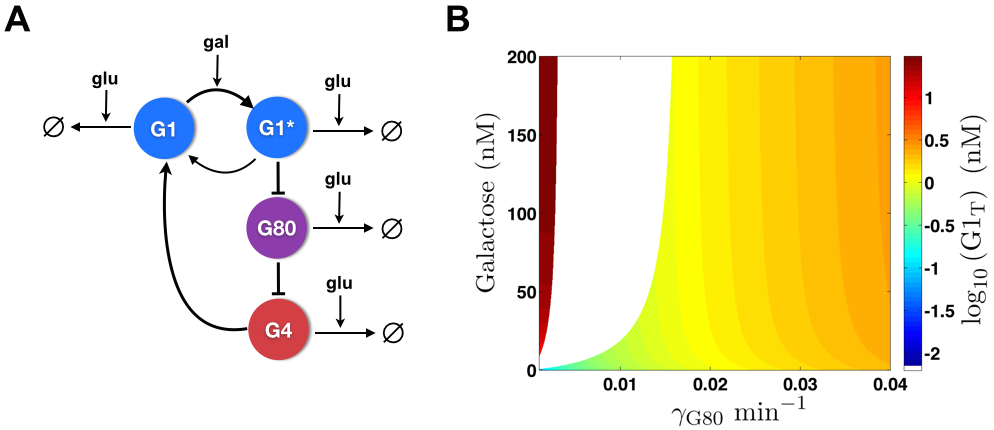

Supplement: S13 Fig — (A) GAL network diagram of the signal transducer Gal1p (G1), repressor Gal80p (G80) and transcription activator Gal4p (G4). In this model, galactose activates the G1 to form G1*, which sequesters G80 from inhibiting G4 by sequestration. The glucose input increases the linear decay rate (dilution rate) of all species in the model by a different scaling factor (see S1 Text). (B) Bifurcation diagram at steady-state. The bifurcation parameters were the degradation rate parameters and galactose. Random parameter sampling (see S1 Text) within physiologically realistic bounds identified a parameter set in which varying the degradation rates in the model can trigger bistability. MATLAB code for panel B can be found in S2 Data and data for panel B can be found in S1 Data. (TIFF) [file pbio.1002042.s015.tiff]

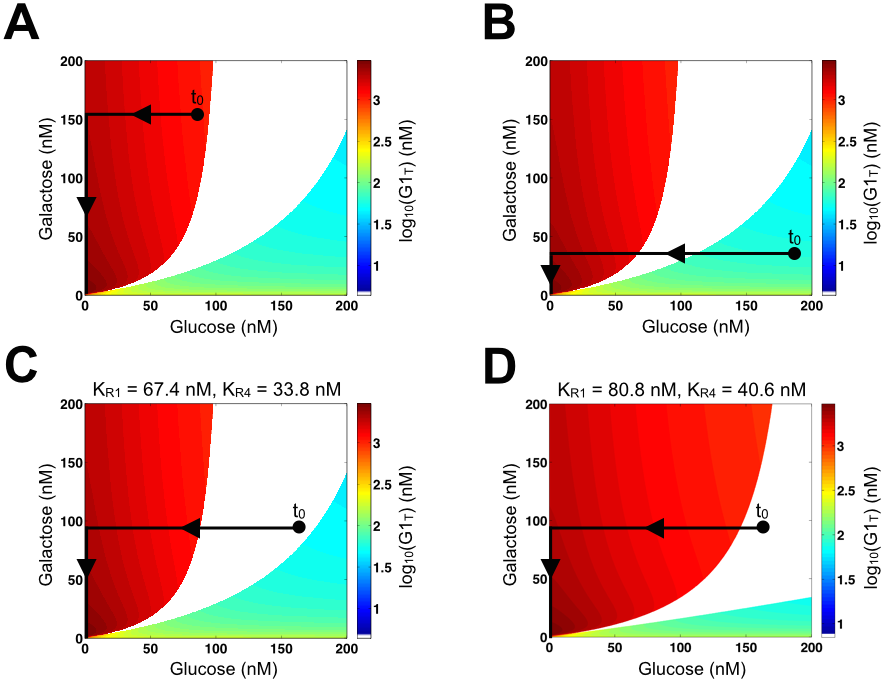

Supplement: S14 Fig — The beginning of the experiment is denoted by t0. The solid line illustrates the sequential consumption of glucose and galactose as a function of time. The colored regions denote monostability and the white region represents bistability. In the colored regions, red indicates monostable ON corresponding to high total G1 levels (G1T) and blue represents monostable OFF corresponding to low G1T. (A) Representative dynamic trajectory in response to galactose and a sufficiently low initial glucose concentration. (B) Representative dynamic trajectory in response to an initial glucose concentration that is significantly higher than the initial galactose level. (C) Representative dynamic trajectory in response to a mixture of glucose and galactose that produces bistability in the model for the parameter set listed in S1 Table. (D) Representative dynamic trajectory in response to the same concentrations of glucose and galactose as (C) for a 20% reduction in the affinity of the glucose-dependent repressor R* to the G1 and G4 promoters (parameter set listed in S1 Table, KR1 = 80.8 nM and KR4 = 40.6 nM). (TIFF) [file pbio.1002042.s016.tiff]

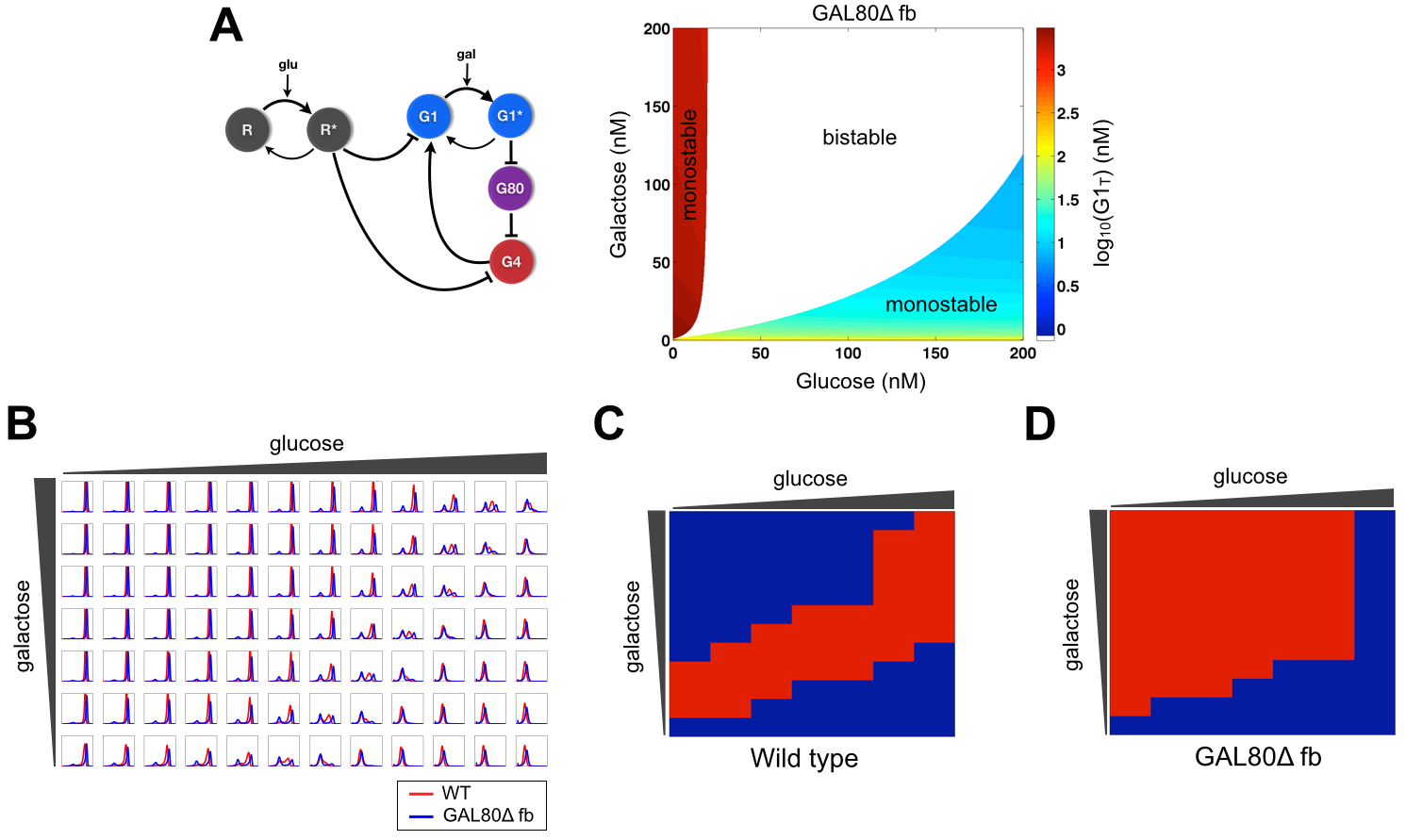

Supplement: S15 Fig — This feature is corroborated by experimental measurements of a strain in which the GAL80 gene is deleted and this gene is expressed from an inducible aTc-responsive promoter in response to 0 ng/ml aTc (GAL80Δ fb). This level of constitutive Gal80p expression corresponds to approximately 40% of fully induced wild type levels [14]. (A) Circuit diagram of the GAL80Δ fb model (left, see S1 Text). Bifurcation diagram of the regions of monostability (colored) and bistability (white) for different values of glucose and galactose (right). The model predicts that removing the GAL80 feedback loop expands the range of glucose and galactose concentrations that produce bistability. (B) Single-cell fluorescence distributions of pGAL10-Venus in the wild type and GAL80Δ fb. (C) Gaussian mixture model (GMM) classification (see Materials and Methods) of experimentally measured pGAL10-Venus gene expression distributions shown in B for the wild type. Red and blue represent a bimodal and monomodal distribution, respectively. (D) GMM classification of experimentally measured fluorescence distributions in B for GAL80Δ fb. Data for panels C and D can be found in S1 Data. (TIFF) [file pbio.1002042.s017.tiff]

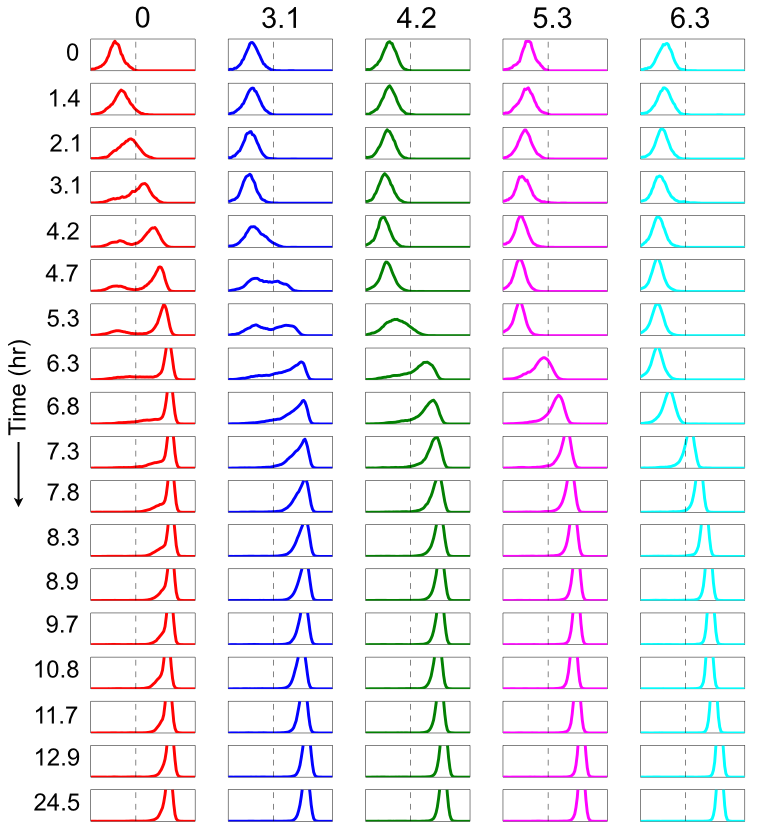

Supplement: S16 Fig — The dotted lines denote the threshold used to compute the fraction of ON cells in Fig. 3. (TIFF) [file pbio.1002042.s018.tiff]

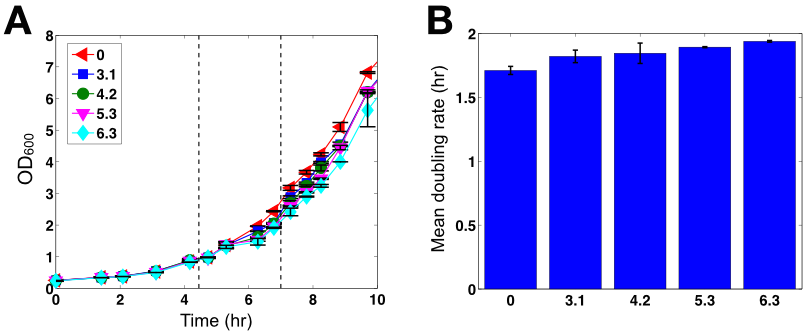

Supplement: S17 Fig — (A) Optical density over time. Dashed lines indicate approximate diauxic shift. (B) Mean doubling rate for each condition during the diauxic shift (dashed lines in A). Data for panels A and B can be found in S1 Data. (TIFF) [file pbio.1002042.s019.tiff]

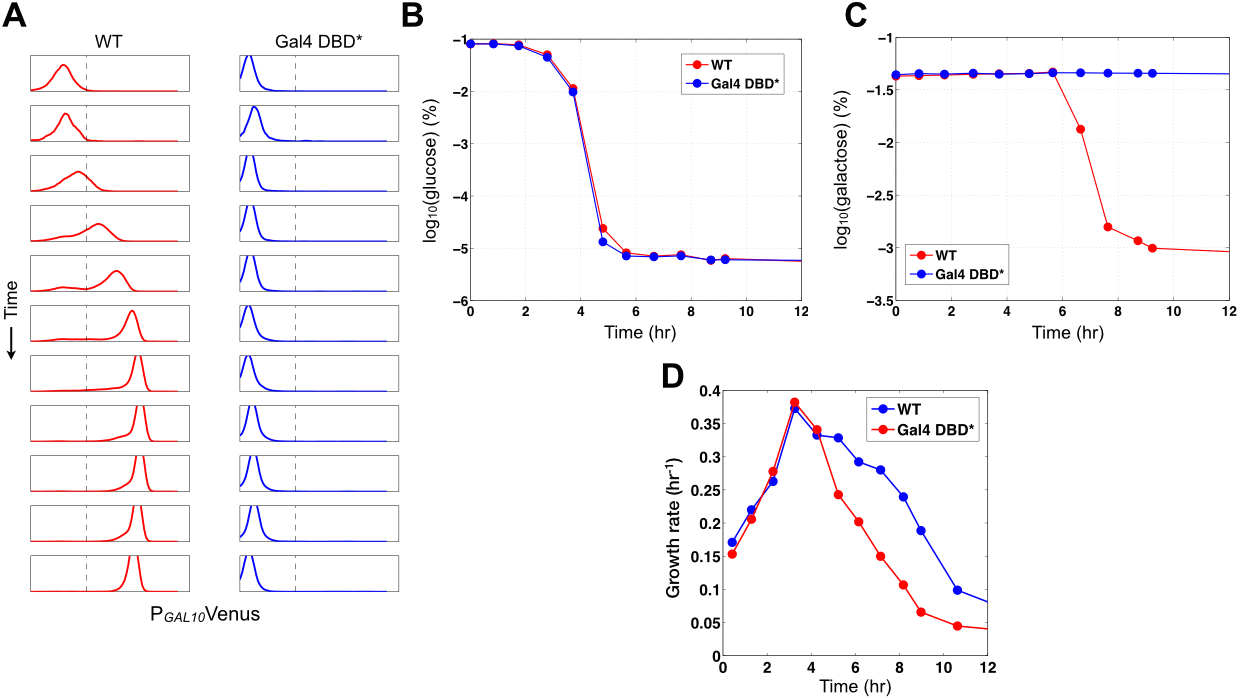

Supplement: S18 Fig — Cells were exposed to 0.1% glucose and 0.1% galactose at the beginning of the experiment. (A) Single-cell fluorescence distributions of a pGAL10-Venus in the wild type and Gal4 DBD*. (B) Glucose concentrations as a function of time. Lines represent fitted Hill functions. (C) Galactose concentrations as a function of time. (D) Growth rates as a function of time. Data for panels B, C, and D can be found in S1 Data. (TIFF) [file pbio.1002042.s020.tiff]

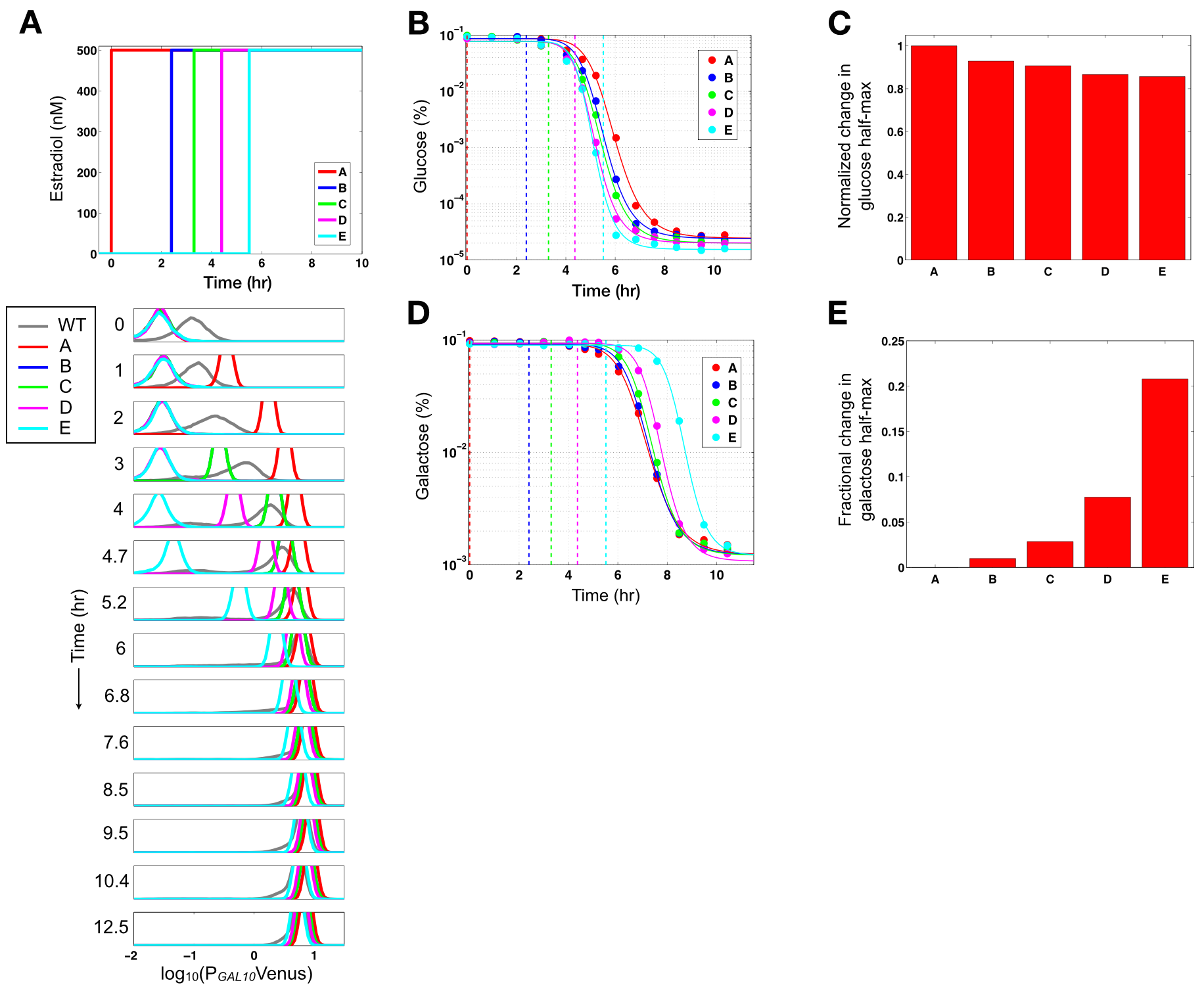

Supplement: S19 Fig — (A) Experimental design. Each condition (A-E) received a step input of 0.1% glucose and 0.1% galactose at time zero and 500 nM estradiol to activate the Gal4 chimera at the indicated times (top). Single-cell fluorescence distributions of pGAL10-Venus as a function of time (bottom). (B) Glucose concentrations as a function of time. Lines represent fitted Hill functions. Dashed lines indicate the time when estradiol was added to each culture. (C) Normalized change in the half-max of the glucose consumption curves for the different conditions relative to condition A. (D) Galactose concentrations as a function of time. (E) Fractional change in the half-max of the galactose concentration curves relative to condition A. Data for panels B, C, D, and E can be found in S1 Data. (TIFF) [file pbio.1002042.s021.tiff]

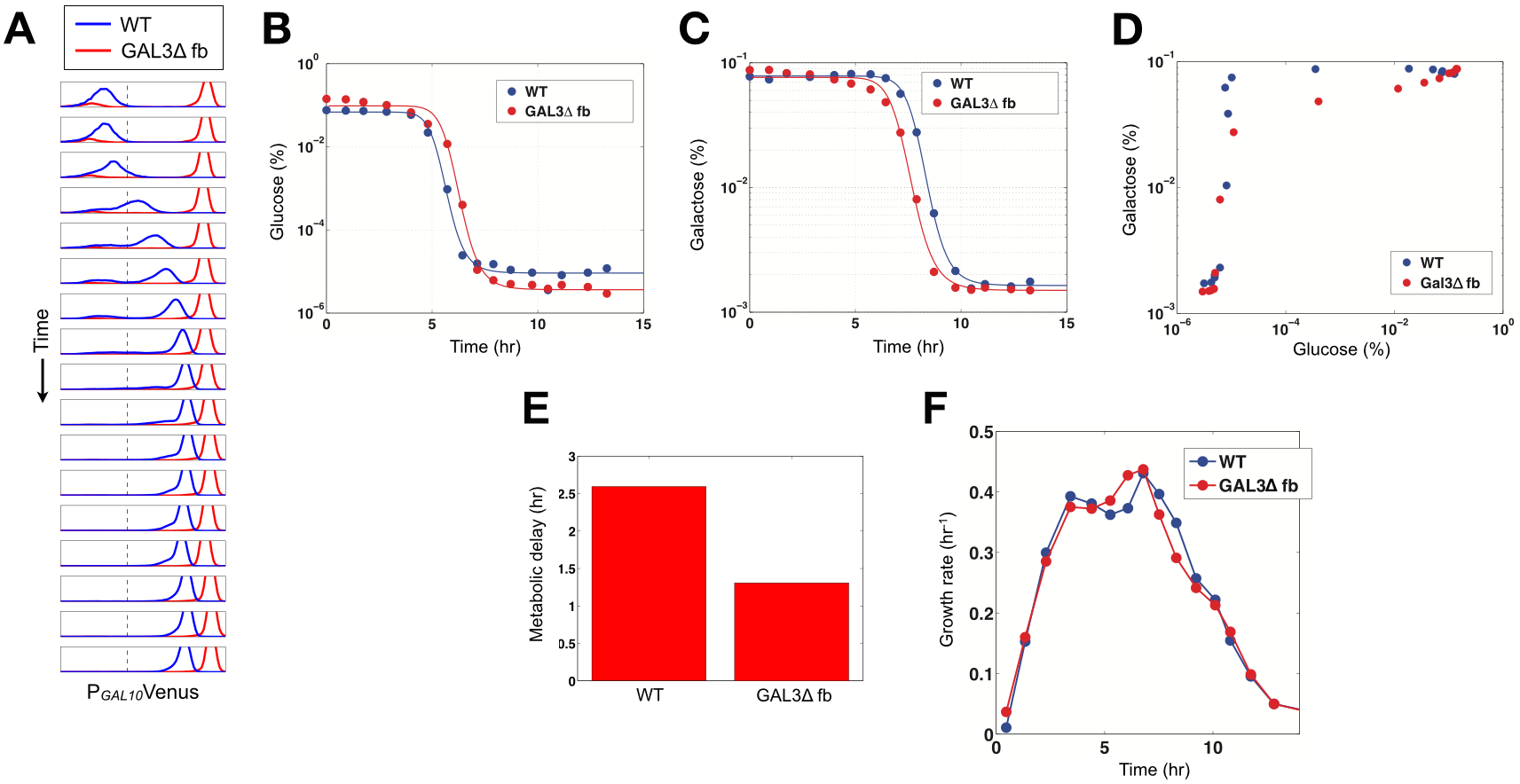

Supplement: S20 Fig — The GAL pathway was induced approximately 12 h in advance of the wild type using a strain in which GAL3 is deleted and this gene is expressed from an inducible aTc-responsive promoter (450 ng/ml aTc, GAL3Δ fb). At time zero, the GAL3Δ fb and wild-type strains received 0.1% glucose and 0.1% galactose. (A) Single-cell fluorescence of pGAL10-Venus in wild type and GAL3Δ fb. (B) Glucose concentrations as a function of time. Lines represent fitted Hill functions. (C) Galactose concentrations as a function of time. (D) Relationship between the glucose and galactose concentrations for the WT and GAL3Δ fb strains. (E) Metabolic delay for wild type and GAL3Δ fb computed by subtracting the half-max of the glucose and galactose curves. (F) Growth rates as a function of time. Data for panels B, C, D, E, and F can be found in S1 Data. (TIFF) [file pbio.1002042.s022.tiff]

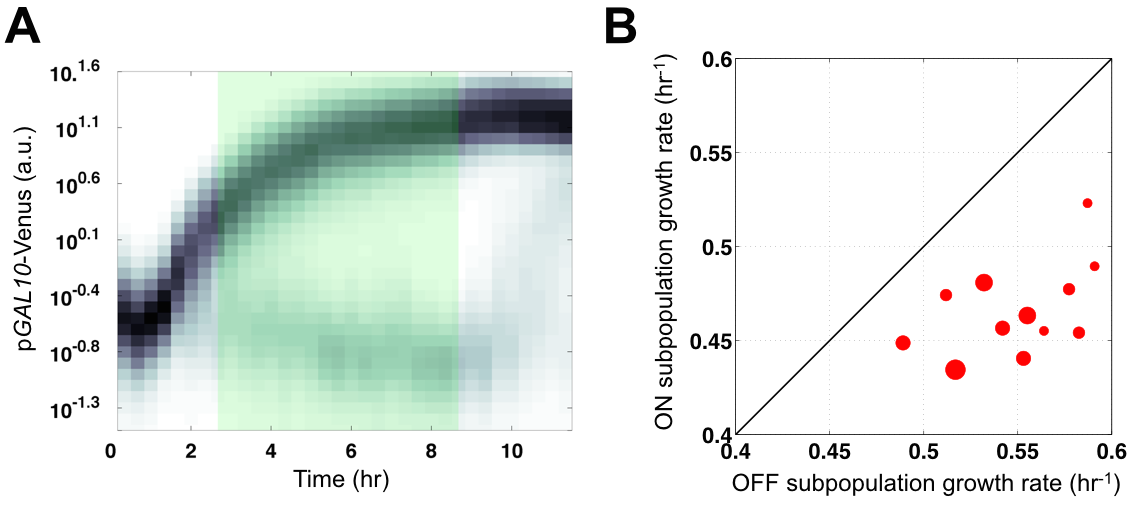

Supplement: S21 Fig — (A) Representative dynamic flow cytometry measurements of pGAL10-Venus in response to 0.5% glucose and 1% galactose from the experiment in Fig. 1. Heat-map of fluorescence distributions as a function of time (left). Quantification of the number of cells in the OFF and ON subpopulations (right, see Materials and Methods). Log2N as a function of time. N represents the number of cells in the GAL ON or OFF subpopulations. (B) Growth rate of GAL OFF subpopulation versus the GAL ON subpopulation for different initial concentrations of glucose and galactose. The diameter of each data point is proportional to the initial concentration of glucose. Data for panel B can be found in S1 Data. (TIFF) [file pbio.1002042.s023.tiff]

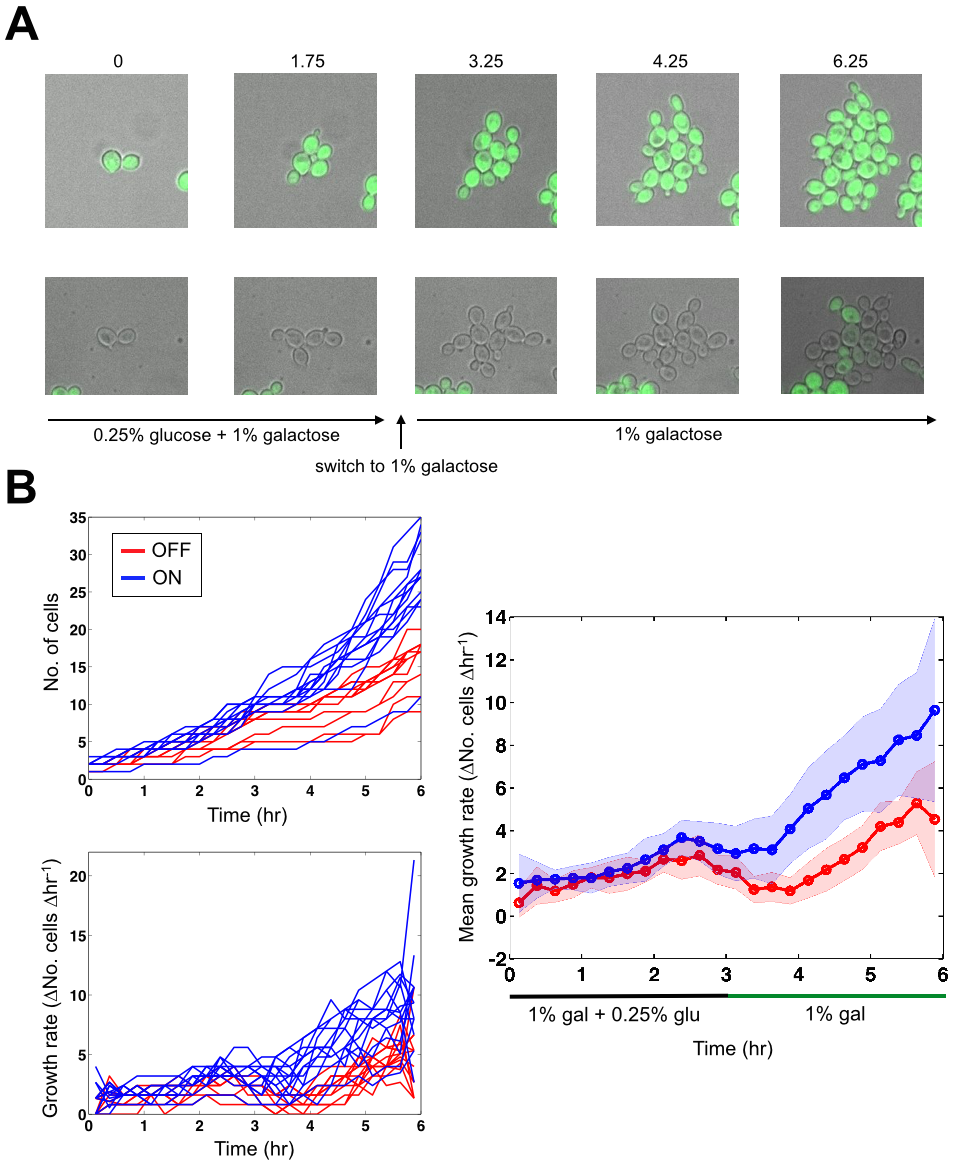

Supplement: S22 Fig — (A) Representative ON (top panels) and OFF (bottom panels) colonies over time. In the microfluidic devices, cells were grown in 1% galactose + 0.25% glucose for 3 h and then switched to 1% galactose media. Numbers indicate when the image was taken in hours. (B) Quantification of the colony growth rates for the ON and OFF subpopulations. Total number of cells over time (top left) for 13 ON or OFF colonies. Growth rate of these colonies over time (bottom left). Mean growth rate over time (right). The shaded regions represent one s.d. from the mean (n = 13). Following the switch to galactose, the difference in the growth rates between the ON and OFF subpopulations is statistically significant with a p-value of 9.8e-04. Data for panels A, B, and C can be found in S1 Data. Data for panel B can be found in S1 Data. (TIFF) [file pbio.1002042.s024.tiff]
