# Supplementary material for: Population Diversification in a Yeast Metabolic Program Promotes Anticipation of Environmental Shifts
Source: PLoS Biol. 2015 Jan 27;13(1):e1002042. doi: 10.1371/journal.pbio.1002042 (PMC4307983; doi:10.1371/journal.pbio.1002042)
Supplement: S1 Text — Section S1.2 describes an ordinary differential equation model with glucose-dependent modification of the decay rates corresponding to S13 Fig. Section S2 reports the modification of the bimodality parameters (δg and FON) in a set of genetic mutants. Section S3 comments on S18 Fig., Section S4 discusses S20 Fig., and Section S5 comments on S21 Fig. (PDF) [file pbio.1002042.s028.pdf]

# Text S1 for

## Population diversification in a metabolic program promotes anticipation of environmental shifts

Ophelia S. Venturelli, Ignacio Zuleta, Richard M. Murray, Hana El-Samad*

*Correspondance to: hana.el-samad@ucsf.edu

# S1 Mathematical modeling

## S1.1 GAL ODE model with glucose repressor

An ODE model of the GAL pathway was constructed based on extensive previous molecular characterization of this system. This model was able to provide explanations for qualitative trends observed in the experimental data and insights into dynamics of the system. The model relied on the following simplifying assumptions:

- No distinction was made between Gal1p and Gal3p since these regulators have the same mechanism for activating the GAL genes by sequestration of Gal80p [1]. We assumed that these two species could be represented by a single species, Gal1p.
- Intracellular transport of glucose through the hexose transporters and galactose through Gal2p was not modeled.
- A glucose repressor, R (for example Mig1) transcriptionally represses *GAL1* and *GAL4* [2].
- Dimerization of Gal4p and Gal80p was not modeled [3, 4].
- We did not differentiate between nuclear and cytoplasmic partitioning of the GAL proteins [5, 6, 7].

The Hill coefficients for G1 ($n_{G1}$, $n_{R1}$), G4 ($n_{R4}$) and G80 ($n_{80}$) were estimated as 3, 2, 1 and 2 [8].
Based on these assumptions, the model that captures a set of molecular interactions for the wild-type (WT) GAL network in the presence of glucose and galactose is

$$\frac{d[\mathrm{G1}]}{dt} = \alpha_{oG1} + \alpha_{G1}\left(\frac{[\mathrm{G4}]^{n_1}}{K_{G1}^{n_1} + [\mathrm{G4}]^{n_1}}\right)\left(\frac{K_{R1}^{n_{R1}}}{K_{R1}^{n_{R1}} + [\mathrm{R}^*]^{n_{R1}}}\right) - k_{fg}[\mathrm{gal}][\mathrm{G1}] + k_{rg}[\mathrm{G1}^*] - \gamma_{G1}[\mathrm{G1}],$$

$$\frac{d[\mathrm{G1}^*]}{dt} = k_{fg}[\mathrm{gal}][\mathrm{G1}] - k_{rg}[\mathrm{G1}^*] - k_{f81}[\mathrm{G1}^*][\mathrm{G80}] + k_{r81}[\mathrm{C81}] - \gamma_{G1s}[\mathrm{G1}^*],$$

$$\frac{d[\mathrm{R}]}{dt} = \alpha_R - k_{fR}[\mathrm{glu}][\mathrm{R}] + k_{rR}[\mathrm{R}^*] - \gamma_R[\mathrm{R}]$$

$$\frac{d[\mathrm{R}^*]}{dt} = k_{fR}[\mathrm{glu}][\mathrm{R}] - k_{rR}[\mathrm{R}^*] - \gamma_{Rs}[\mathrm{Rs}]$$

$$\frac{d[\mathrm{G4}]}{dt} = \alpha_{G4}\left(\frac{K_{R4}^{n_{R4}}}{K_{R4}^{n_{R4}} + [\mathrm{R}^*]^{n_{R4}}}\right) - k_{f84}[\mathrm{G4}][\mathrm{G80}] + k_{r84}[\mathrm{C84}] - \gamma_{G4}[\mathrm{G4}],$$

$$\frac{d[\mathrm{G80}]}{dt} = \alpha_{oG80} + \alpha_{G80}\left(\frac{[\mathrm{G4}]^{n_{80}}}{K_{G80}^{n_{80}} + [\mathrm{G4}]^{n_{80}}}\right) - k_{f81}[\mathrm{G1}^*][\mathrm{G80}] + k_{r81}[\mathrm{C81}] - k_{f84}[\mathrm{G4}][\mathrm{G80}] + k_{r84}[\mathrm{C84}] - \gamma_{G80}[\mathrm{G80}],$$

$$\frac{d[\mathrm{C81}]}{dt} = k_{f81}[\mathrm{G1}^*][\mathrm{G80}] - k_{r81}[\mathrm{C81}] - \gamma_{C81}[\mathrm{C81}],$$

$$\frac{d[\mathrm{C84}]}{dt} = k_{f84}[\mathrm{G4}][\mathrm{G80}] - k_{r84}[\mathrm{C84}] - \gamma_{C84}[\mathrm{C84}],$$

where G1, G4 and G80 represent Gal1p, Gal4p and Gal80p. We assume that the the concentrations of the complexes, G1-gal (G1*), R-glu (R*), Gal1p-Gal80p (C81) and Gal4p-Gal80p (C84) are at quasi-steady-state with respect to the dynamics of G1, R, G4 and G80. Using this assumption, we can simplify the system of equations above to the following four ODEs

$$\frac{d\mathrm{G1}}{dt} = \alpha_o - \gamma_{G1}\mathrm{G1} - k_{fg}\mathrm{G1gal} + \frac{\mathrm{G1}k_{rg}p_3}{p_4 + p_5\mathrm{G80}} + \frac{\alpha_{G1}K_{R1}^2\mathrm{G4}^3}{\left(K_{R1}^2 + \mathrm{R}^2p_2^2\right)\left(K_{G1}^3 + \mathrm{G4}^3\right)},$$

$$\frac{d\mathrm{R}}{dt} = \alpha_R - \gamma_R\mathrm{R} - k_{fR}\mathrm{gluR} + k_{rR}p_2\mathrm{R},$$

$$\frac{d\mathrm{G80}}{dt} = \alpha_{oG80} - \gamma_{G80}\mathrm{G80} - \frac{p_6\mathrm{G1G80}}{p_4 + p_5\mathrm{G80}} + p_7\mathrm{G80G4} + \frac{\alpha_{G80}\mathrm{G4}^2}{K_{R4}^2 + \mathrm{G4}^2},$$

$$\frac{d\mathrm{G4}}{dt} = \frac{\alpha_{G4}K_{R4}}{K_{R4} + p_2\mathrm{R}} - \gamma_{G4}\mathrm{G4} + p_8\mathrm{G80G4}.$$

where $p_0 = \frac{k_{fs}}{\gamma_{C81}+k_{rs}}$, $p_1 = \frac{k_{f84}}{\gamma_{C84}+k_{r84}}$, $p_2 = \frac{\text{glu}k_{fR}}{\gamma_{Rs}+k_{rR}}$, $p_3 = k_{fg}\text{gal}$, $p_4 = \gamma_{G1s} + k_{rg}$, $p_5 = k_{f81} - k_{r81}p_0$, $p_6 = k_{f81}p_3 + k_{r81}p_0p_3$, $p_7 = p_1k_{r84} - k_{f84}$ and $p_8 = p_1k_{r84} - k_{f84}$.
At steady-state, $\frac{d[\text{G1}]}{dt} = \frac{d[\text{R}]}{dt} = \frac{d[\text{G4}]}{dt} = \frac{d[\text{G80}]}{dt} = 0$ and we could solve for the equilibrium concentrations of G1, R and G4 in terms of G80. The equilibrium value of G80, $\text{G80}_e$ was determined by computing the roots of a ninth order polynomial

$$a_0 + a_1\text{G80}_e + \cdots + a_9\text{G80}_e^9 = 0,$$

The parameter values for this model are listed in Table S1.
where the coefficients, $a_i$, are functions of the model parameters. The *GAL80* feedback deletion model was constructed by replacing the Hill function of G4 by a constant $\alpha_{G80s}$. The equilibrium points of this model were determined by computing the roots of a sixth order polynomial of $\text{G80}_e$. The stability of the equilibrium points was determined by computing the eigenvalues of the Jacobian matrix of the system of equations evaluated at each equilibrium point [9].

## S1.2 GAL ODE model with glucose-dependent modification of decay rates

In this model, we assume that the dominant effect of glucose is indirect by modifying the cellular growth rate. A change in growth rate due to glucose corresponds to a change in the linear decay rates of the protein species in the GAL network model. In this case, the decay rate and galactose concentrations are bifurcation parameters. The equations for this model include

$$\frac{d[\text{G1}]}{dt} = \alpha_{oG1} + \alpha_{G1}\left(\frac{[\text{G4}]^{n_1}}{K_{G1}^{n_1} + [\text{G4}]^{n_1}}\right) - k_{fg}[\text{gal}][\text{G1}] + k_{rg}[\text{G1}^*] - \gamma_{G1}[\text{G1}],$$
$$\frac{d[\text{G1}^*]}{dt} = k_{fg}[\text{gal}][\text{G1}] - k_{rg}[\text{G1}^*] - k_{f81}[\text{G1}^*][\text{G80}] + k_{r81}[\text{C81}] - \gamma_{G1s}[\text{G1}^*],$$
$$\frac{d[\text{G4}]}{dt} = \alpha_{G4} - k_{f84}[\text{G4}][\text{G80}] + k_{r84}[\text{C84}] - \gamma_{G4}[\text{G4}],$$
$$\frac{d[\text{G80}]}{dt} = \alpha_{oG80} + \alpha_{G80}\left(\frac{[\text{G4}]^{n_{80}}}{K_{G80}^{n_{80}} + [\text{G4}]^{n_{80}}}\right) - k_{f81}[\text{G1}^*][\text{G80}] + k_{r81}[\text{C81}] - k_{f84}[\text{G4}][\text{G80}] + k_{r84}[\text{C84}] - \gamma_{G80}[\text{G80}],$$
$$\frac{d[\text{C81}]}{dt} = k_{f81}[\text{G1}^*][\text{G80}] - k_{r81}[\text{C81}] - \gamma_{C81}[\text{C81}],$$
$$\frac{d[\text{C84}]}{dt} = k_{f84}[\text{G4}][\text{G80}] - k_{r84}[\text{C84}] - \gamma_{C84}[\text{C84}].$$

Similarly to the method described above, we assume that the the concentrations of the complexes, G1-gal ($\text{G1}^*$), Gal1p-Gal80p (C81) and Gal4p-Gal80p (C84) are at quasi-steady-state with respect to the dynamics of G1, G4 and G80. Using this assumption, we can simplify the system of equations above to the following four ODEs

$$\frac{d[\text{G1}]}{dt} = \alpha_{oG1} - \gamma_{G1}[\text{G1}] - k_{fg}[\text{G1}][\text{gal}] + \frac{\alpha_{G1}[\text{G4}]^3}{[\text{G4}]^3 + K_{G1}^3} + \frac{a_1k_{rg}[\text{G1}]}{a_2 + a_3[\text{G80}]},$$
$$\frac{d[\text{G4}]}{dt} = \alpha_{G4} - \gamma_{G4}[\text{G4}] - k_{f84}[\text{G4}][\text{G80}] + \frac{k_{f84}k_{r84}}{a_5}[\text{G4}][\text{G80}],$$
$$\frac{d[\text{G80}]}{dt} = \alpha_{oG80} - \gamma_{G80}[\text{G80}] - \frac{a_1k_{f81}[\text{G1}][\text{G80}]}{a_2 + a_3[\text{G80}]} - k_{f84}[\text{G4}][\text{G80}] + \frac{\alpha_{G80}[\text{G4}]^2}{[\text{G4}]^2 + K_{\text{G80}}^2} + \frac{a_1k_{r81}k_{f81}\text{G1G80}}{a_4(a_2 + a_3[\text{G80}])} + \frac{k_{f84}k_{r84}}{a5}[\text{G4}][\text{G80}],$$

where $a_1 = [\text{gal}]k_{fg}(\gamma_{C81} + k_{r81})$, $a_2 = \gamma_{C81}\gamma_{G1s} + \gamma_{G1s}k_{r81} + \gamma_{C81}k_{rg} + k_{r81}k_{rg}$, $a_3 = \gamma_{C81}k_{f81}$, $a_4 = \gamma_{C81} + k_{r81}$, $a_5 = \gamma_{C84} + k_{r84}$. At steady-state, $\frac{d[\text{G1}]}{dt} = \frac{d[\text{G4}]}{dt} = \frac{d[\text{G80}]}{dt} = 0$ and we could solve for the equilibrium concentrations of G1 and G4 in terms of G80. The equilibrium value of G80, $\text{G80}_e$ was determined by computing the roots of a ninth order polynomial

$$a_0 + a_1\text{G80}_e + \cdots + a_9\text{G80}_e^9 = 0.$$

Since a change in growth rate should modulate the decay rates of all proteins, we first assumed that all decay rate parameters should be equal as they were varied. Latin Hypercube Sampling was used to scan 100 values of each parameter over a broad and physiologically plausible range. Using this approach, no parameter sets were identified that were able to recapitulate the bistability region.

We next introduced some flexibility by allowing each of the degradation rate parameters to be different. To do so, we assumed that $\gamma_{G1} = c_{G1}\gamma_{G80}$, $\gamma_{G1s} = c_{G1s}\gamma_{G80}$, $\gamma_{G4} = c_{G4}\gamma_{G80}$, $\gamma_{C81} = c_{C81}\gamma_{G80}$ and $\gamma_{C84} = c_{C84}\gamma_{G80}$ where $c_{G1}$, $c_{G1s}$, $c_{G4}$, $c_{C81}$ and $c_{C84}$ represent scaling parameters. Using Latin Hypercube Sampling, a parameter set was identified that generated a region of bistability that qualitatively matches the region of bimodality observed in our experimental data (Figure S13B and parameter values are listed in Table S2). Our results showed that the G80 degradation rate had to be high for the system to operate in a monostable OFF regime corresponding to high glucose concentrations.

# S2 Modification of $\delta_g$ and $F_{\text{ON-mid}}$ in a set of mutants

Glucose repression is a complex process that modifies the activity of the GAL pathway at both the transcriptional and post-transcriptional levels [10]. Transcriptional repression of the GAL genes is mediated by a set of DNA binding proteins that recruit the global transcriptional repression complex Cyc8-Tup1, modifying the promoter state using multiple mechanisms including chromatin remodeling [11, 2, 12, 13, 14]. In response to galactose, the GAL promoters can escape a repressed state by the liberation of Gal4p from Gal80p and the inhibition of Cyc8-Tup1, which then initiates an ordered recruitment of the SAGA complex and TBP [15]. A PHD domain protein, Cti6p, has also been shown to play an important role in overcoming the transcriptional repression of the *GAL1* promoter by Cyc8-Tup1 [16].

In addition, the bifunctional glucose kinase, Hxk2p, also has been shown to play a critical role in glucose repression by regulating the nuclear to cytoplasmic ratio of Mig1p by blocking its phosphorylation by the Snf1 kinase [17, 18]. Hxk2p has a glucose-dependent nuclear localization and physically interacts with Mig1p to regulate a set of target genes [19, 20, 21]. Furthermore, a previous study showed that a deletion of *HXK2* disrupts the hierarchical consumption order of glucose and galactose [22]. Interestingly, the recruitment of Cyc8-Tup1 to the *GAL1* promoter, as opposed to the changes in localization of Mig1, has been shown to be the dominant regulatory interaction that forces this system into a repressed state [23].

To gain mechanistic insight into the role of glucose repression in generating the pre-emptive galactose pathway bimodal strategy (Fig. 1), we characterized a set of mutants that modify the transcriptional state of the GAL promoters. For this experiment, we started with similar cell densities for each mutant ($OD_{600}$ $\approx$ 0.3). Our results showed that a deletion of *CTI6* significantly extended $\delta_g$. Indeed, there were cells in the repressed subpopulation that never turned on the GAL genes over a period of approximately 14 hours (Figure S8A-1). We also found that the fraction of cells in the ON state at the midpoint of the transient bimodal region ($F_{\text{ON-mid}}$) was significantly lower in the *CTI6*$\Delta$ strain compared to WT (Figure S8D). These data suggest that the timing of the repressed state's activation depends on CTI6 at the promoter-level.

Interestingly, deletion of the dominant glucose kinase, Hxk2, significantly extended $\delta_g$ compared to WT (Figure S8A-2). Similar to the *CTI6*$\Delta$ cells, the cells in the repressed subpopulation did not switch to the ON state during the course of the experiment. This mutant was particularly intriguing since it was the only modification to the system in which both $F_{\text{ON-mid}}$ and $\delta_g$ were augmented (Figure S8C,D). Previous results have shown that cells deleted for Hxk2p consume glucose and galactose simultaneously [22]. Taken together, we hypothesize that Hxk2p is a crucial player that links the availability of glucose to the repression of galactose consumption and timing of GAL gene induction.

We next characterized the dynamics of a strain in which Gal80p was regulated by an inducible *TET* promoter as opposed to its endogenous Gal4p-dependent promoter, therefore abolishing the feedback regulation of this protein (GAL80$\Delta$ fb) [8]. In the presence of 19.7 ng/ml aTc, the repressed subpopulation did not induce the GAL genes over the course of the experiment (Figure S8A-3). These data demonstrate that a high and unregulated concentration of Gal80p relative to WT can block the repressed population from inducing the GAL genes at the appropriate time, highlighting the sensitivity of the network to perturbations in the regulation of this repressor.

We also identified three mutants that displayed smaller $\delta_g$ phenotypes compared to WT. The *MIG1* binding site is degenerate and other transcription factors can bind to the same sequence [24]. We hypoth-

esized that a gene deletion of *MIG1* could increase the probability that these alternative transcriptional regulators bind to the putative *MIG1* sites and thus potentially alter the expression level of target genes. To directly reduce the promoter-level repression without significantly perturbing promoter occupancy, four point mutations were introduced into Mig1 ($\text{Mig1}_{4m}$ contains L490A, L493A, L496A and L498A) that have been shown to reduce the affinity of Mig1 to the Cyc8-Tup1 complex [25, 23, 26]. This mutant exhibited a smaller $\delta_g$ and a larger $F_{\text{ON-mid}}$ compared to WT (Figure S8B-1,C,D).

We examined the role of the Cyc8-Tup1 complex in the regulation of the transient bimodal dynamics. Since a deletion the Cyc8-Tup1 complex has severe pleiotrophic effects, including flocculation, diploids were constructed that contain only one allele of *CYC8* and *TUP1* and therefore half the dosage of this complex. We compared the dynamics of this mutant to a wild type diploid and observed a significantly smaller $\delta_g$ and larger $F_{\text{ON-mid}}$ compared to wild type (Figure S8B-2,C,D).

To measure the contribution of Gal80p-Gal4p sequestration on the dynamics of the system, the affinity of Gal80p to Gal4p was reduced by mutating F856C or M861C of *GAL4* [27]. The F856C mutation reduces the affinity of Gal80 to Gal4 more significantly than M861C. These two mutants exhibited a significantly smaller $\delta_g$ than WT (Figure S8B-3,C). F856C had a smaller $\delta_g$ compared to M861C, suggesting that the binding affinity of the Gal80p-Gal4p complex is a crucial variable that controls the duration of bimodality. In addition, these two mutants exhibited a larger $F_{\text{ON-mid}}$ compared to WT (Figure S8D).

We also measured the growth rates of these strains to determine if the observed changes in the system's dynamics could be explained by alterations in the growth rates and thus the consumption rate of glucose. However, the changes in $\delta_g$ and $F_{\text{ON-mid}}$ were significantly greater than the variation in the growth rates (Figure S8E). Therefore, these data strongly suggest that the observed modifications of the transient bimodality phenotype were dominated by the perturbations to the system's regulation.

Taken together, these results suggest that the timing of the delayed activation response and the bias in the fraction of ON cells is regulated in a distributed way by many factors. Future investigation will reveal how these multiple players combine to generate the observed dynamic phenotypes. However, our data indicate that promoter-level regulation plays a critical role in controlling these phenotypes.

# S3 Galactose does not benefit cells until glucose is consumed

To assess the contribution of galactose to growth in the presence of glucose, we measured the extracellular concentration of glucose, galactose, growth and GAL gene expression dynamics of a strain lacking the endogenous *GAL4* gene and expressing a DNA binding mutant of *GAL4* (C14Y). This mutant renders Gal4p unable to activate transcription of the GAL genes [28] (Figure S18A). Our data showed that this mutant consumed glucose at a rate equivalent to WT but was unable to consume galactose (Figure S18B,C). Furthermore, the growth rates of the mutant and WT were very similar while the cells were consuming glucose (Figure S18D). These data demonstrate that the presence of galactose does not significantly alter the growth rate of the cell population until glucose is consumed.

# S4 Constitutive GAL gene expression reduces the glucose consumption rate

Our data indicated that a high expression level of the GAL genes in a subpopulation of cells was not sufficient to initiate galactose metabolism (Fig. 2). To test whether altering GAL gene expression can modify the timing of galactose metabolism in the presence of glucose we used a strain in which the endogenous *GAL3* gene was deleted and Gal3p was expressed from an inducible *TET* promoter regulated by an aTc-responsive transcription factor rtTA (GAL3Δ fb) [8, 29]. Using this strain, we induced the GAL system in advance of the wild type by administering 450 ng/ml aTc and monitored the dynamics of sugar consumption following induction of these two strains with 0.1% glucose and 0.1% galactose (Figure S20A).

The GAL3Δ fb strain exhibited an approximately 10% diminished rate of glucose consumption compared to wild type, indicating that constitutive GAL gene expression can have an inhibitory effect on glucose metabolism (Figure S20B). Prior to glucose depletion, the mutant slowly consumed a small amount of galactose and galactose metabolism was accelerated in the mutant relative to WT (Figure S20C). These

combined effects reduced the metabolic delay, defined as the difference in the half-max of the galactose and glucose curves, by approximately 50% compared to WT (Figure S20D). The GAL3Δ fb mutant displayed an enhanced growth rate relative to WT during the diauxic shift and a reduced growth rate relative to WT following the diauxic shift (Figure S20F).

# S5 Growth rates of the ON and OFF subpopulations

For many dual-sugar inputs, the OFF and ON subpopulations were clearly distinguishable for several hours. We assumed that the transitions between the two states were insignificant since cellular switching between the OFF and ON states would yield intermediate fluorescence levels due to the gradual accumulation or dilution of fluorescent proteins and stochastic switching was not detected in time-lapse microscopy experiments (Figure S4A,B). During this period, we computed the growth rates of the individual subpopulations as described in the Materials and Methods. Our results showed that the GAL OFF subpopulation grew on average 15% faster than the GAL ON subpopulation (Figure S21). Since our data has shown that the glucose consumption rate can be reduced by GAL gene expression (Figure S19 and S20), this effect could explain the diminished growth rate of the GAL OFF population in these conditions.

## References


[1] Bhat P, Hopper J (1992) Overproduction of the GAL1 or GAL3 protein causes galactose-independent activation of the GAL4 protein: evidence for a new model of induction for the yeast GAL/MEL regulon. Molecular Cell Biology 12: 2701-2707.

[2] Nehlin J, Carlberg M, Ronne H (1991) Control of yeast GAL genes by MIG1 repressor: a transcriptional cascade in the glucose response. EMBO J 10: 3373-7.

[3] Hong M, Fitzgerald M, Harper S, Luo C, Speicher D, et al. (2008) Structural basis for dimerization in DNA recognition by Gal4. Structure 16: 1019-26.

[4] Pilauri V, Bewley M, Diep C, Hopper J (2005) Gal80 dimerization and the yeast GAL gene switch. Genetics 169: 1903-14.

[5] Peng G, Hopper J (2000) Evidence for Gal3p's cytoplasmic location and Gal80p's dual cytoplasmic-nuclear location implicates new mechanisms for controlling Gal4p activity in *Saccharomyces cerevisiae*. Molecular Cellular Biology 20: 5140-8.

[6] Wightman R, Bell R, Reece R (2008) Localization and interaction of the proteins constituting the GAL genetic switch in *Saccharomyces cerevisiae*. Eukaryotic Cell 7: 2061-8.

[7] Egriboz O, Jiang F, Hopper J (2011) Rapid GAL gene switch of *Saccharomyces cerevisiae* depends on nuclear Gal3, not nucleocytoplasmic trafficking of Gal3 and Gal80. Genetics 189: 825-36.

[8] Venturelli O, El-Samad H, Murray R (2012) Synergistic dual positive feedback loops established by molecular sequestration generate robust bimodal response. Proceedings of the National Academy of Sciences 109.

[9] Aström K, Murray R (2008) Feedback Systems: An Introduction for Scientists and Engineers. Princeton University Press.

[10] Gancedo J (1998) Yeast carbon catabolite repression. Microbiology Molecular Biology Review 62: 334-61.

[11] Zhou H, Winston F (2001) NRG1 is required for glucose repression of the SUC2 and GAL genes of *Saccharomyces cerevisiae*. BMC Genetics 2: 5.

[12] Wong K, Struhl K (2011) The Cyc8-Tup1 complex inhibits transcription primarily by masking the activation domain of the recruiting protein. Genes and Development 25: 2525-39.

[13] Smith R, Johnson A (2000) Turning genes off by Ssn6-Tup1: a conserved system of transcriptional repression in eukaryotes. Trends in Biochemical Sciences 25: 325-30.

[14] Keleher C, Redd M, Schultz J, Carlson M, Johnson A (1992) Ssn6-Tup1 is a general repressor of transcription in yeast. Cell 68: 709-19.

[15] Larschan E, Winston F (2001) The *S. cerevisiae* SAGA complex functions in vivo as a coactivator for transcriptional activation by Gal4. Genes and Development 15: 1946-56.

[16] Papamichos-Chronakis M, Petrakis T, Ktistaki E, Topalidou I, Tzamarias D (2002) Cti6, a PHD domain protein, bridges the Cyc8-Tup1 corepressor and the SAGA coactivator to overcome repression at GAL1. Molecular Cell 9: 1297-305.

[17] Ahuatzi D, Riera A, Pelaez R, Herrero P, Moreno F (2007) Hxk2 regulates the phosphorylation state of Mig1 and therefore its nucleocytoplasmic distribution. Journal of Biological Chemistry 282: 4485-93.

[18] Ma H, Botstein D (1986) Effects of null mutations in the hexokinase genes of *Saccharomyces cerevisiae* on catabolite repression. Molecular Cellular Biology 6: 4046-52.

[19] Randez-Gil F, Herrero P, Sanz P, Prieto J, Moreno F (1998) Hexokinase pii has a double cytosolic-nuclear localisation in *Saccharomyces cerevisiae*. FEBS Letters 425: 475-8.

[20] Ahuatzi D, Herrero P, de la Cera T, Moreno F (2004) The glucose-regulated nuclear localization of hexokinase 2 in *Saccharomyces cerevisiae* is Mig1-dependent. Journal of Biological Chemistry 279: 14440-6.

[21] Fernandez-Garcia P, Pelaez R, Herrero P, Moreno F (2012) Phosphorylation of yeast hexokinase 2 regulates its nucleocytoplasmic shuttling. Journal of Biological Chemistry 287: 42151-64.

[22] Raamsdonk L, Diderich J, Kuiper A, van Gaalen M, Kruckeberg A, et al. (2001) Co-consumption of sugars or ethanol and glucose in a *Saccharomyces cerevisiae*strain deleted in the HXK2 gene. Yeast 18: 1023-33.

[23] Papamichos-Chronakis M, Gligoris T, Tzamarias D (2004) The Snf1 kinase controls glucose repression in yeast by modulating interactions between the Mig1 repressor and the Cyc8-Tup1 co-repressor. EMBO Reports 5: 368-72.

[24] Wu J, Trumbly R (1998) Multiple regulatory proteins mediate repression and activation by interaction with the yeast Mig1 binding site. Yeast 14: 985-1000.

[25] Ostling J, Cassart J, Vandenhaute J, Ronne H (1998) Four hydrophobic amino acid residues in the c-terminal effector domain of the yeast mig1p repressor are important for its in vivo activity. Molecular Genetics and Genomics 260: 269-79.

[26] Treitel M, Carlson M (1995) Repression by SSN6-TUP1 is directed by MIG1, a repressor/activator protein. Proceedings of the National Academy of Sciences 92: 3132-6.

[27] Ansari A, Reece R, Ptashne M (1998) A transcriptional activating region with two contrasting modes of protein interaction. Proceedings of the National Academy of Sciences 95: 13543-8.

[28] Johnston M, Dover J (1987) Mutations that inactivate a yeast transcriptional regulatory protein cluster in an evolutionarily conserved DNA binding domain. Proceedings of the National Academy of Sciences 84: 2401-5.

[29] Urlinger S, Baron U, Thellmann M, Hasan M, Bujard H, et al. (2000) Exploring the sequence space for tetracycline-dependent transcriptional activators: novel mutations yield expanded range and sensitivity. Proceedings of the National Academy of Sciences of the United States of America 97: 7963-8.
